# Inhibition of *Atg7* in intestinal epithelial cells drives resistance against *Citrobacter rodentium*

**DOI:** 10.1038/s41419-025-07422-5

**Published:** 2025-02-19

**Authors:** David Cune, Caterina Luana Pitasi, Alessia Rubiola, Trinath Jamma, Luca Simula, Camille Boucher, Apolline Fortun, Lucie Adoux, Franck Letourneur, Benjamin Saintpierre, Emmanuel Donnadieu, Benoît Terris, Pascale Bossard, Benoît Chassaing, Béatrice Romagnolo

**Affiliations:** 1 Université Paris Cité, Institut Cochin, Inserm, CNRS, Paris, France; 2 Equipe Labellisée Ligue Nationale Contre Le Cancer, Paris, France; 3 Department of Biological Sciences, Birla Institute of Technology and Science-Pilani, Hyderabad, India; 4 Genomic Facility, Université de Paris Cité, Institut Cochin, INSERM, CNRS, Paris, France; 5 Pathology Department, AP-HP, Hôpital Cochin, Paris, France; 6 Microbiome-Host Interactions, Institut Pasteur, Université Paris Cité, INSERM, Paris, France

**Keywords:** Infection, Acute inflammation

## Abstract

Autophagy, a cytoprotective mechanism in intestinal epithelial cells, plays a crucial role in maintaining intestinal homeostasis. Beyond its cell-autonomous effects, the significance of autophagy in these cells is increasingly acknowledged in the dynamic interplay between the microbiota and the immune response. In the context of colon cancer, intestinal epithelium disruption of autophagy has been identified as a critical factor influencing tumor development. This disruption modulates the composition of the gut microbiota, eliciting an anti-tumoral immune response. Here, we report that Atg7 deficiency in intestinal epithelial cells shapes the intestinal microbiota leading to an associated limitation of colitis induced by *Citrobacter rodentium* infection. Mice with an inducible, intestinal epithelial-cell-specific deletion of the autophagy gene, Atg7, exhibited enhanced clearance of *C. rodentium*, mitigated hyperplasia, and reduced pathogen-induced goblet cell loss. This protective effect is linked to a higher proportion of neutrophils and phagocytic cells in the early phase of infection. At later stages, it is associated with the downregulation of pro-inflammatory pathways and an increase in Th17 and Treg responses—immune responses known for their protective roles against *C. rodentium* infection, modulated by specific gut microbiota. Fecal microbiota transplantation and antibiotic treatment approaches revealed that the Atg7-deficiency-shapped microbiota, especially Gram-positive bacteria, playing a central role in driving resistance to *C. rodentium* infection. In summary, our findings highlight that inhibiting autophagy in intestinal epithelial cells contributes to maintaining homeostasis and preventing detrimental intestinal inflammation through microbiota-mediated colonization resistance against *C. rodentium*. This underscores the central role played by autophagy in shaping the microbiota in promoting immune-mediated resistance against enteropathogens.

## Introduction

Intestinal homeostasis involves reciprocal communication between the host and gut microbiota, which plays a crucial role in the immune response. Indeed, the composition of the microbiota significantly influences the outcome of intestinal diseases by shaping the host’s immune response. Notably, gut dysbiosis is recognized as a critical factor in inflammatory bowel disease [1]. Furthermore, mounting evidence suggests that the gut microbiota plays a pivotal role in both the development and treatment response of colon cancer, influencing the immune landscape of the tumor microenvironment [2–4]. Therefore, gaining a deeper understanding of the intricate interplay between the host, microbiota, and immune response is imperative for shedding light on the etiology of various intestinal diseases and exploring novel avenues for preventive and therapeutic strategies.

Autophagy, a catabolic process, is crucial for intracellular quality control, recycling damaged organelles, eliminating aggregates and misfolded macromolecules. It also maintains cellular homeostasis under various stress by providing metabolic nutrients during starvation, and acting as a defense mechanism against microbial attacks [5]. Numerous studies have demonstrated the role of autophagy in intestinal homeostasis [6]. Our previous investigations, using mice deficient in the essential autophagy gene Atg7 in intestinal epithelial cells (IEC), revealed that basal autophagy protects small intestine stem cells from intrinsic stress signals such as DNA damage or ROS accumulation, as well as specific microbial signals like muramyl dipeptide [7]. Dysregulated autophagy is linked to intestinal diseases such as inflammatory bowel disease and colorectal cancer. Genome-wide association studies associate autophagy genes with a higher risk of IBD. However, mice lacking key autophagy genes like Atg7, Atg5, or Atg16L1 in IEC do not show reduced lifespan, suggesting that a deficiency in autophagy alone is not enough to cause chronic intestinal inflammation [7–10]. Autophagy also plays an important role in intestinal tumor development and therapy [2, 11]. We showed that Atg7 was required for both tumor initiation and intestinal tumor cell metabolism and that the loss of Atg7 in IEC prevents tumor initiation by shaping an antitumoral immune response linked to a change in the composition of the microbiota [2]. Interestingly, multiple other studies have shown that deficiency in autophagy genes alter the gut microbiota [2, 12–14]. Thus, autophagy in the intestinal epithelium emerges as a regulator of both the gut microbiota composition and the immune response, two crucial players in combating pathogenic bacteria.

*C. rodentium* is a well-characterized murine bacterial pathogen that causes similar pathology to human enteropathogenic *Escherichia coli* [15]. These Gram-negative bacteria provoke transient colitis by inducing attaching and effacing lesions on the intestinal epithelium. Mounting an effective defense against *C. rodentium* relies on signals from commensal microbes, as evidenced by higher *C. rodentium* levels and impaired infection clearance in germ-free and antibiotic-treated mice [16]. The role of autophagy during *C. rodentium* infection remains contentious with the overall impact contingent on context and the underlying mechanism have to be further explored [17–19].

In our study, we report that inhibiting autophagy through Atg7 deletion in IEC confers resistance against *C. rodentium*. This resistance is achieved by suppressing the inflammatory response, limiting colonic hyperplasia, and preventing goblet cell loss. Atg7-deficient mice exhibit higher proportions of neutrophils and phagocytic cells in the early phase of infection. This is followed by reduced lymphocyte recruitment but a higher proportion of Th17 and Treg in the colonic lamina propria at later stage—two crucial lymphocyte subsets promoting a protective immune response to *C. rodentium*. This protection mediated by ATG7 loss is dependent on the intestinal microbiota, and more specifically Gram-positive bacteria, and can be transmitted through fecal microbiota transfer.

## Material and methods

### Ethical compliance and statement

All animal procedures were conducted in compliance with French legal regulations (Ministère de la Recherche, de l’Enseignement Supérieur et de l’Innovation) and received approval from the Ethics Committee of Paris University (APAFIS 8722 and 15557).

### Mice

Atg7^fl/fl^VilCre^ERT2^ have been described previously [7]. The control mice were their Cre-negative littermates (Atg7^fl/fl^VilCre^ERT2−^). All experiments were conducted on the C57Bl/6 background and under conventional housing conditions. Mice were provided ad libitum access to a standard laboratory diet. Tamoxifen injections were administered to mice aged 2–3 months. Both male and female mice were included in the study, with intraperitoneal injections of 1 mg tamoxifen per day for 5 consecutive days.

### Antibiotic and vancomycin treatment

For antibiotic treatment, we administered a combination of ampicillin (1 g L^−1^), neomycin (1 g L^−1^), metronidazole (1 g L^−1^), and vancomycin (0.5 g L^−1^) with an additional 1.5% saccharose in drinking water for one week. In the case of vancomycin treatment, 0.5 g L^−1^ of vancomycin was added to drinking water for a duration of 13 days. The antibiotic solution was refreshed three times a week. Subsequent to the completion of the antibiotic protocol, mice were provided ad libitum access to regular drinking water for the remainder of the experiment.

### Fecal microbiota transfer

Fecal microbiota transfer was conducted three days following the completion of antibiotic treatments. A total of 150 mg of feces from donor mice was utilized per recipient mouse. The fecal samples were obtained from untreated, age-matched donor mice with Atg7^ΔIEC^. Stools were pooled, homogenized in a phosphate-buffered saline solution (PBS), and centrifuged to pellet the particulate matter, and the supernatant then collected. Subsequently, 200 µl of the fresh stool supernatant was administered to WT mice via oral gavage for three consecutive days.

### *Citrobacter rodentium* infection and monitoring

The *C. rodentium* strain ICC180 was cultured overnight in Luria-Bertani broth supplemented with kanamycin at 37 °C in a shaking incubator [20]. An oral administration of 5 × 10^9^ colony-forming units (cfu) in 200 µl of PBS was given to each mouse. Mouse weights were recorded every 2 days. For the quantification of bacterial burden, stools were collected, weighed, homogenized in PBS, and plated in serial dilution on Luria-Bertani plates supplemented with kanamycin. In vivo imaging was conducted using the IVIS Spectrum CT system from PerkinElmer.

### Histology, immunochemistry, and immunofluorescence

Immediately following euthanasia, the entire gastrointestinal tract of the mouse was carefully extracted, opened along its length, and rolled from the proximal to distal end to create a “Swiss roll.” The tissues were fixed by incubating them in 4% paraformaldehyde overnight at 4 °C and subsequently embedded in paraffin wax. Sections of 3 μm thickness were cut and stained with hematoxylin and eosin. For immunohistochemistry, following a previously described protocol [7], 5-μm sections were treated with 3% hydrogen peroxide for 15 min at room temperature. In both immunohistochemistry and immunofluorescence analyses, antigen retrieval was performed by boiling sections for 15 min in citrate buffer (10 mM, pH 6) using a microwave pressure cooker. Sections were then incubated in a blocking solution (1% bovine serum albumin (BSA) for MPO staining, or 1% BSA and 2% normal goat serum for other stainings) for 20 min.

The sections were then incubated for 1 h at room temperature or overnight at 4 °C with primary antibodies, appropriately diluted in blocking solution. Primary antibodies targeting the following proteins were employed: Ki67 (12202S, 1:200; Cell Signaling), CD3 (ab5690, 1:200; Abcam), CD4 (ab6640, 1:400; Abcam), F4/80 (ab183685, 1:200; Abcam), P-Stat3 (9131L, 1:100; Cell signaling) MPO (AF3667, 1:200 Bio-Techne), Foxp3 (14-5773-82, 1:100; Invitrogen), and Rorγt (14-6988-82, 1:100; Invitrogen). For immunohistochemistry, specific binding was detected using a biotinylated secondary antibody and ABC reagent (vector). The signal was subsequently revealed with a 3,3′-diaminobenzidine tetrahydrochloride solution (Vector), with hematoxylin serving as the counterstain. For immunofluorescence, Alexa Fluor-coupled secondary antibodies (Thermo Fisher Scientific) were used, and Hoechst was added for nuclei counterstaining. Finally, slides were mounted using mowiol. The images were captured with the Olympus BX63F wide-field microscope equipped with Olympus software. For MPO, CD4, and CD3 analysis, in each section, positive cells were counted and lamina propria area was measured. Results were expressed as the relative amount of total positive cells related to the total area of the lamina propria.

### Mucus layer preservation

Immediately following euthanasia, 1 cm of the proximal colon was taken and divided into 3. Tissues were immediately fixed in Carnoy’s fixative solution for 48 h and subsequently embedded in paraffin wax. Sections of 5 μm thickness were cut and stained with alcian blue. Fast red was added for counterstaining. Images were obtained using a Lamina Slide Scanner from Akoya Perkin Elmer.

### Flow cytometric analyses

Immune cells were isolated from the lamina propria. The colon was rinsed in cold PBS, minced into small pieces, and incubated with EDTA (2 mmol/L) followed by digestion with collagenase (1.5 mg/ml) for 30 min at 37 °C. A discontinuous Percoll separation method (40% and 80%) was employed to purify immune cells. Cell suspensions were centrifuged, and the pellet was resuspended in 40% Percoll, overlaid by 80% Percoll (Cytiva). Cells concentrated at the interface were collected, washed in cold PBS, and then resuspended in RPMI1640 containing 5% fetal bovine serum. Following cell counting, the lymphocytes were incubated with a human Fc receptor binding inhibitor polyclonal antibody (14-9161-73; eBioscience) and subsequently stained with specific antibodies.

Cells underwent fluorescence-activated cell sorting (FACS) analysis using the following antibodies: anti-CD4 (RM4-5 clone, 558107; BD Biosciences), anti-TCRβ (H57-597, 109224; Biolegend), anti-CD19 (1D3 clone, 550992; BD Biosciences), anti-FOXP3 (FJK-16s clone, 560220; BD Biosciences), anti-IL-10 (JES5-16E3 clone, 554467; BD Biosciences), anti-RORγt (REA278 clone, 130-123-248; Miltenyi Biotec), and anti-IL-17A (TC11-18H10 clone, 560220; BD Biosciences), anti-CD45 (HI30 clone, 304024; Biolegend), anti-CD11c (N418 clone, 117334; Biolegend), anti-CD11b (M1/70 clone, 101251; Biolegend), anti-Ly6C (HK1.4 clone, 128026; Biolegend), anti-CD172a (P84 clone, 144008; Biolegend), anti-CD64 (X57/7.1 clone, 139311; Biolegend), anti-CD86 (GL-1 clone, 105040; Biolegend), anti-CD44 (IM7 clone, 103041, Biolegend), anti-Ly6G (1A8 clone, 127608; Biolegend), anti-MHC II (M5/114.15.2, 107645; Biolegend), anti-F4/80 (CI:A3-1 clone, MCA497A647T; Biorad). Dead cells were excluded using the LIVE/DEAD Fixable Aqua Dead Cell Stain Kit (L34957, Invitrogen) or Zombie UV Fixable Viability Dye (Biolegend 423108).

For intracellular staining of lymphocytes, cells were pretreated with phorbol myristate acetate/ionomycin for 4 h at 37 °C, 5% CO2, and brefeldin A was added for the last 3 h. Following this, cells were stained with specific extracellular antibodies, permeabilized using BD cytoFix/cytoPerm solution (555028; BD Biosciences) or with the transcription factor staining buffer set (00-5523-00; eBioscience), and finally stained intracellularly. FACS analysis was conducted using the BD LSR Fortessa flow cytometer (BD Biosciences) and analyzed with FlowJo software (BD Biosciences) and FACSDiva software (BD Biosciences).

### Ex vivo organ culture

Colon tissue was harvested from mice, opened longitudinally, and washed in PBS twice. 0.5 cm biopsies from the distal colon were weighed and cultured in complete medium (RPMI1640 (Thermofisher) supplemented with 100 U/mL of penicillin-streptomycin (Thermofisher), 50 μg/mL gentamicin (Thermofisher) and 10% FBS) for 24 h at 37 °C. IL-1β, IL-6, and TNFα in the supernatants collected at day 0, day 8 and day 18 p.i and IL-22 supernatant collected at day 0 were measured by ELISA using U-Plex Assay kit (MSD). Results were normalized according to the weight of the biopsies.

### Sialic acid measuring

Fecal samples from mice were collected and frozen at −80 °C. A total of 10 mg of feces per mouse was reconstituted in distilled water (100 mg/ml) and homogenized. Supernatant was collected after centrifugation for 15 min at 14,000 × *g* and used to quantify free sialic acid with the QuantiChrom™ Sialic Acid Assay Kit (Bioassay system) according to the manufacturer’s protocol.

### RNA/DNA extraction and quantitative real-time PCR analysis

Total RNA was extracted from distal colonic mouse tissues using Trizol reagent (Invitrogen). Reverse transcription was performed using 1 µg of total RNA with the Maxima First-Strand cDNA Synthesis Kit. DNA extraction from mice feces was carried out using the QIAamp DNA Stool Mini Kit (Qiagen). Quantitative RT-PCR was conducted on a Light Cycler 480 thermocycler (Roche) and specific primers (Supplementary Table 1). Gene expression levels and bacterial abundance were quantified using the 2^(−Delta Ct)^ method, with 18 s and 16 s rRNA expressions serving as internal controls, respectively.

### RNA-seq analysis

Distal colon RNA was extracted using Trizol reagent following the manufacturer’s protocol for RNA sequencing. A minimum of three mice per genotype was included in the RNA-seq analysis. Library preparation and sequencing were conducted by the Genom’IC platform at Institut Cochin. Libraries were prepared using the TruSeq Stranded messenger RNA protocol from Illumina, starting with 800 ng high-quality total RNA (RNA Integrity Number >8). Paired-end sequencing (2 × 75 bp) was performed on an Illumina Nextseq 500 platform, utilizing 20–40 million paired-end reads for subsequent analysis.

After sequencing, initial analysis involved AZOAN software (ENS, Paris) for demultiplexing and quality control on raw data (utilizing FastQC modules, version 0.11.5). The STAR algorithm (version 2.7.6a; Genom’IC) was then employed to align Fastq files with the Ensembl Mus musculus GRCm38 reference, release 101 (Genom’IC). Subsequently, reads were counted using RSEM (v1.3.1; Genom’IC), and statistical analyses were performed on the read counts using R (version 3.6.3; Genom’IC) to identify the proportion of genes differentially expressed between two conditions.

The DESeq2 normalization method (DESeq2 median of ratios with DESeq function) was applied, with a prefilter of reads and genes (reads uniquely mapped on the genome or up to 10 different loci with a count adjustment, and genes with at least 10 reads in at least 3 different samples). Following recommendations for this package, the Wald test with the contrast function and the Benjamini–Hochberg false discovery rates control procedure were employed to identify Differentially Expressed Genes (DEGs). R scripts and parameters are accessible on GitHub (https://github.com/BSGenomique/genomic-Rnaseq-pipeline/releases/tag/v1.0420).

### Microbiota analysis by 16S rRNA gene sequencing using Illumina technology

16S rRNA gene amplification and sequencing were performed using the Illumina MiSeq technology following the protocol described previously [21, 22]. The 16S rRNA genes, region V4, were PCR amplified from each sample using a composite forward primer and a reverse primer containing a unique 12-base barcode, designed using the Golay error-correcting scheme, which was used to tag PCR products from respective samples [21]. The forward primer 515F was used: 5’*AATGATACGGCGACCACCGAGATCTACACGC*TXXXXXXXXXXXX**TATGGTAATT*GT***GTGYCAGCMGCCGCGGTAA-3′: the italicized sequence is the 5′ Illumina adapter, the 12 × sequence is the Golay barcode, the bold sequence is the primer pad, the italicized and bold sequence is the primer linker and the underlined sequence is the conserved bacterial primer 515F. The reverse primer 806R used was :5’*CAAGCAGAAGACGGCATACGAGAT***AGTCAGCCAG*CC***GGACTACNVGGGTWTCTAAT-3′: the italicized sequence is the 3′ reverse complement sequence of Illumina adapter, the bold sequence is the primer pad, the italicized and bold sequence is the primer linker and the underlined sequence is the conserved bacterial primer 806R. PCR reactions consisted of 5PRIME HotMasterMix (Quantabio, Beverly, MA, USA), 0.2 μM of each primer, 10–100 ng template, and reaction conditions were 3 min at 95 °C, followed by 30 cycles of 45 s at 95 °C, 60 s at 50 °C and 90 s at 72 °C on a Biorad thermocycler. PCR products were visualized by gel electrophoresis and purified with Ampure magnetic purification beads (Agencourt, Brea, CA, USA). Products were then quantified (Quant-iT PicoGreen dsDNA assay), and a master DNA pool was generated from the purified products in equimolar ratios. The pooled products were quantified using the Quant-iT PicoGreen dsDNA assay and sequenced using an Illumina MiSeq sequencer (paired-end reads, 2 × 250 bp) at the Genom’IC sequencing platform from Institut Cochin, Paris, France.

### 16S rRNA gene sequence analysis

16S rRNA sequences were analyzed using QIIME2 – version 2022 [23]. Sequences were demultiplexed and quality filtered using Dada2 method [24] with QIIME2 default parameters in order to detect and correct Illumina amplicon sequence data, and a table of Qiime 2 artifact was generated. A tree was next generated, using the align-to-tree-mafft-fasttree command, for phylogenetic diversity analyses, and alpha and beta diversity analysis were computed using the core-metrics-phylogenetic command. For taxonomy analysis, features were assigned to operational taxonomic units (OTUs) with a 99% threshold of pairwise identity to the Greengenes reference database version 13.8 [25].

### Software analysis

Transcriptomics data analyses were conducted utilizing Gene Set Enrichment Analysis (GSEA) and ClueGo software. Heatmaps were generated using the Genesis and Venn diagrams were created using the Bioinformatics and Evolutionary Genomics tool (https://bioinformatics.psb.ugent.be/webtools/Venn/).

### Statistical analysis

Statistical analyses were performed with GraphPad Prism (9.04). Graphical data are presented as means ± SEM. Data were checked for normality, and statistical analyses were performed using unpaired two-tailed Student’s *t*-tests or two-way ANOVA with Geisser-Greenhouse correction. *p*-values below 0.05 were considered statistically significant. The significance levels are denoted as follows: *p < 0.05, **p < 0.01, ***p < 0.001. No statistical methods were used to pre-determine sample sizes, but our sample sizes are similar to those generally employed in the field. A minimum of *n* = 3 animals was systematically analyzed. Data collection and analysis were not performed in a blinded manner. The experiments were not randomized.

## Results

### *Atg7* deletion in IEC confers protection against *C. rodentium* infection

**Fig. 1:**
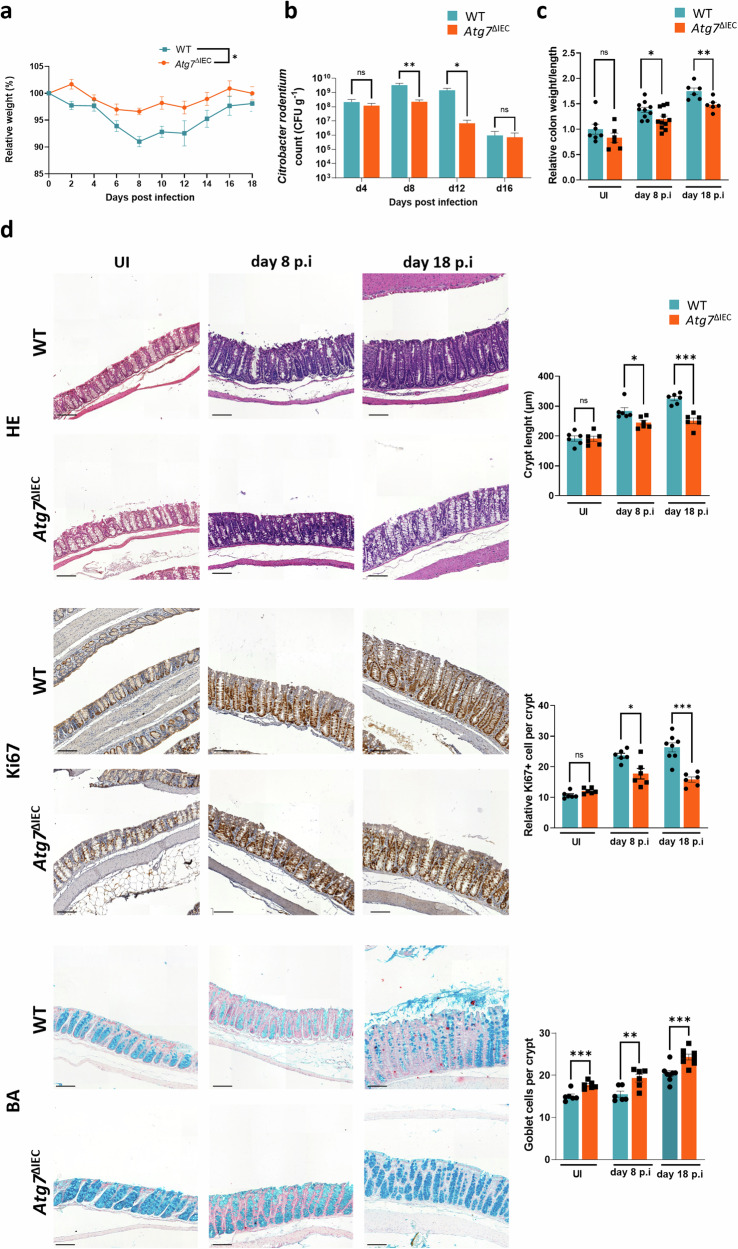
*Atg7* deletion in colonic epithelium protects against *C. rodentium* infection. **a** Body weight changes in WT and *Atg7*^ΔIEC^ mice after oral infection with *C. rodentium* (*n* = 9 WT and *n* = 10 *Atg7*^ΔIEC^ from two independent experiments). Two-way ANOVA with Greenhouse–Geisser correction. **b** Quantification of bacterial burden in stool (CFU/g) over time from WT and *Atg7*^ΔIEC^ mice (*n* = 6 WT and *n* = 8 *Atg7*^ΔIEC^ from two independent experiments). **c** Relative quantification of colon weight/length ratio from WT and *Atg7*^ΔIEC^ mice before (uninfected, UI) and after infection at day 8 and day 18 p.i (UI: n = 7 WT, *n* = 6 *Atg7*^ΔIEC^; at day 8 p.i: *n* = 10 WT, *n* = 11 *Atg7*^ΔIEC^; and at day 18 p.i: *n* = 6 WT, *n* = 6 *Atg7*^ΔIEC^ mice from two independent experiments). **d** Histological analysis of WT and *Atg7*^ΔIEC^ colonic epithelium after *C. rodentium* infection. Representative hematoxylin & eosin (H&E), Ki67 and alcian blue (BA) staining on colonic sections from WT and *Atg7*^ΔIEC^ mice before and after infection at day 8 and day 18 p.i. Scale bar = 100 µm. Quantification of colonic crypts length (*n* = 6 mice per condition from two independent experiments), Ki67 positive cells per crypts (UI: *n* = 6 WT, *n* = 6 *Atg7*^ΔIEC^, at day 8: *n* = 6 WT and *n* = 6 *Atg7*^ΔIEC^, at day 18 p.i: *n* = 8 WT and *n* = 6 *Atg7*^ΔIEC^ day 18 p.i. from two independent experiment) and BA-positive cells per crypts (UI: *n* = 6 WT mice, *n* = 6 *Atg7*^ΔIEC^ mice; at day 8 p.i: *n* = 6 WT mice, *n* = 6 *Atg7*^ΔIEC^ mice and at day 18 p.i: *n* = 8 WT mice, *n* = 6 *Atg7*^ΔIEC^ mice from two independent experiments). Significant differences: *p < 0.05, **p < 0.01, ***p < 0.001, ns not significant, determined by unpaired *t*-test. Mean ± SEM.

To investigate the role played by autophagy during *C. rodentium* infection, we used our previously established *Atg7*^fl/fl^*VillinCreER*^T2^ mouse model to induce deletion of Atg7 specifically in IEC and *Atg7*^fl/fl^ mice as control mice [7]. Both mutant and control mice were subjected to tamoxifen injections; henceforth, they are referred to as Atg7^ΔIEC^ and WT mice, respectively. We monitored bacterial colonization and host health status following oral gavaging of *Atg7*^ΔIEC^ and WT mice with 5 × 10^9^ bioluminescent *C. rodentium* strain ICC180 incubator [20]. *Atg7*^ΔIEC^ mice exhibit reduced body weight loss (Fig. 1a) compared to WT mice. In the first day post-infection, the pathogen primarily resides in the cecum, corresponding to the establishment phase before colonization of the colonic mucosa and the expansion phase. Accordingly, at day 4 post-infection (p.i) the bacterial load in stool was not significantly different between WT and *Atg7*^ΔIEC^ mice (Fig. 1b). However, after the expansion phase of *C. rodentium*, we noticed a higher bacterial burden in WT mice than in *Atg*7^ΔIEC^ mice at day 8 and 12 p.i (Fig. 1b). Consistently, in vivo bioluminescent imaging depicted a lower *C. rodentium* signal in *Atg7*^ΔIEC^ mice than in their WT counterpart 8 days p.i (Fig. S1a). Morphometric analysis of the colon demonstrated that infected WT mice displayed a higher colon weight/length ratio at day 8 p.i (active stage of the infection) and day 18 p.i (recovery phase) compared to infected *Atg7*^ΔIEC^ mice (Fig. 1c). The decreased susceptibility of *Atg7*^ΔIEC^ mice to *C. rodentium* infection were validated by histological analysis. Microscopic analysis of WT colonic tissues at day 8 and day 18 p.i revealed characteristic features of *C. rodentium*-induced colitis, marked by significant crypt elongation due to pronounced hyperproliferation (Fig. 1d). These histological damages were notably attenuated in Atg7^ΔIEC^ mice (Fig. 1d). Importantly, such differences were absent at steady state between WT and Atg7^ΔIEC^ mice (Fig. 1d). Consistent with prior reports, the crypt hyperplasia observed in WT mice at day 8 and day 18 p.i was associated with loss of mature cells (Fig. 1d). Quantification of alcian blue-positive cells at steady state and during infection in *Atg7*^ΔIEC^ mice revealed a greater number of goblet cells in Atg7^ΔIEC^ mice compared to WT mice (Fig. 1d). These findings indicate that intestinal ATG7 is crucial for maintaining goblet cells both under steady-state conditions and during *C. rodentium* infection. This is consistent with previous studies, which showed that partial autophagy deficiency in *Atg5*^*+/−*^ mice leads to pronounced goblet cell hyperplasia [26]. However, this hyperplasia is not accompanied by an increased mRNA expression of goblet cell markers (Fig. S2a). Additionally, we observed a thinner mucus layer in *Atg7*^*ΔIEC*^ mice, aligning with studies suggesting that autophagy regulates mucus secretion from colonic goblet cells (Fig. S2b) [21, 22]. Altogether, these results indicate that *Atg7* deletion throughout the intestinal epithelium contributes to the host protection against *C. rodentium* infection.

### Role of *Atg7* in the inflammatory response to *C. rodentium* infection

**Fig. 2:**
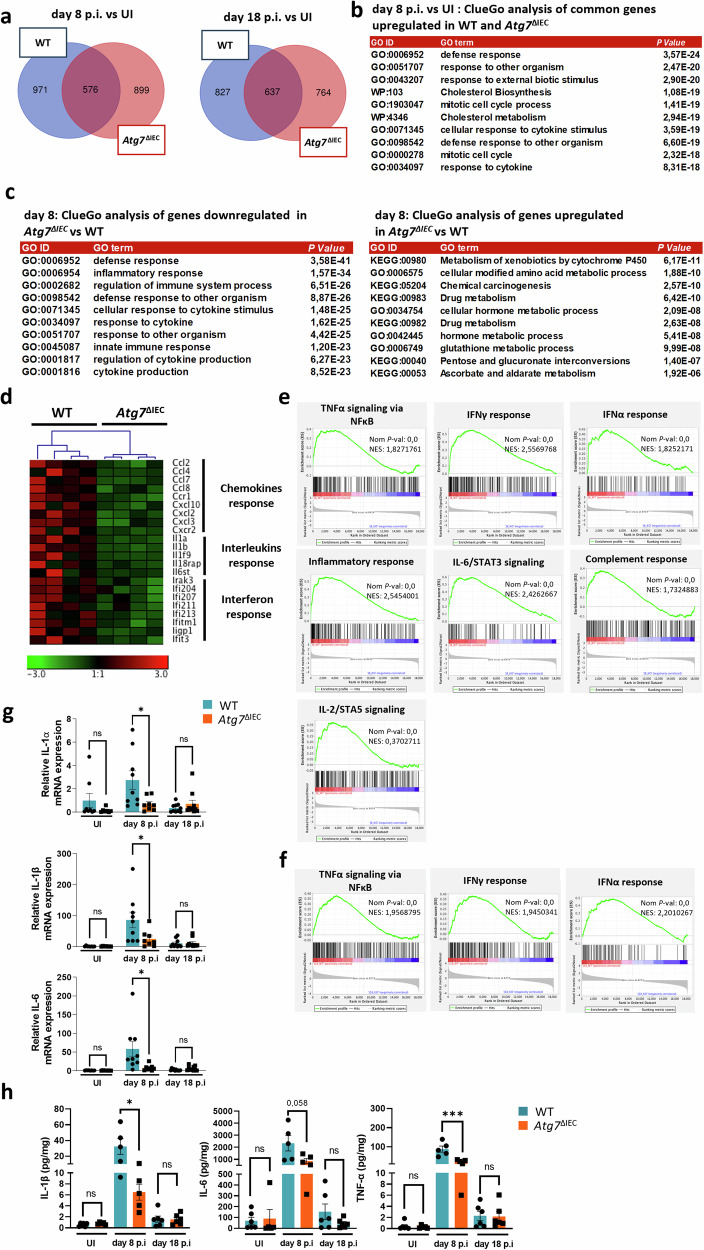
Atg7 deficiency alters the immune transcriptional response during *C. rodentium* infection. RNA-seq analysis of whole colonic tissues from WT and *Atg7*^ΔIEC^ mice was conducted before (UI) and after infection at day 8 and day 18 p.i (at UI: *n* = 3 WT mice, *n* = 3 *Atg7*^ΔIEC^ mice; at day 8 p.i: *n* = 4 WT mice, n = 4 *Atg7*^ΔIEC^ mice; at day 18 p.i: *n* = 5 WT mice, *n* = 5 *Atg7*^ΔIEC^ mice). **a** Venn diagrams displaying the number of genes with significantly increased expression on day 8 and day 18 in WT, *Atg7*^ΔIEC^, or both, compared to their non-infected counterpart (UI). **b** ClueGO analysis of the top ten biological pathways of genes commonly upregulated in WT and *Atg7*^ΔIEC^ colons compared to UI of the same genotype at day 8 p.i. **c** ClueGO analysis of the top ten biological pathways of up and downregulated genes in *Atg7*^ΔIEC^ colon compared to WT at day 8 p.i. **d** Heatmap displaying the expression values of genes associated with interleukin, chemokine, and interferon in colonic sections of WT and *Atg7*^ΔIEC^ at day 8 p.i based on RNA-seq data. **e** Enrichment plot generated by GSEA for hallmark TNFα signaling via NFKB, IFNγ response, IFNα response, inflammatory response, IL-6/Stat3 signaling, complement response, and IL-2/Stat5 genes sets based on RNA-seq data from colonic tissues of WT and *Atg7*^ΔIEC^ mice at day 8 p.i. **f** Enrichment plot generated by GSEA for hallmark TNFα signaling via NFKB, IFNγ response and IFNα response genes sets based on RNA-seq data from colonic tissues of WT and *Atg7*^ΔIEC^ mice at day 18 p.i. **g** Assessment of relative IL-1α, IL-1β and IL-6 mRNA levels by qRT-PCR on colonic sections of WT and *Atg7*^*Δ*IEC^ mice before and after infection at day 8 and day 18 p.i (UI: *n* = 8 WT mice, *n* = 7 *Atg7*^ΔIEC^ mice; at day 8 p.i: *n* = 9 WT mice; *n* = 8 Atg7^ΔIEC^ mice; at day 18 p.i: *n* = 10 WT mice, *n* = 10 *Atg7*^ΔIEC^ mice from two independent experiments). **h** Elisa analysis for IL-1β, IL-6, and TNFα secretion by colon explant cultures of WT and *Atg7*^ΔIEC^ mice before (UI) and after *C. rodentium* infection (UI: *n* = 6 WT mice, *n* = 5 *Atg7*^ΔIEC^; day 8 p.i: *n* = 5 WT mice, *n* = 5 *Atg7*^ΔIEC^ mice; day 18 p.i: *n* = 5 WT mice, *n* = 5 *Atg7*^ΔIEC^). Significant difference: *p < 0.05, ***p < 0.001, ns not significant, determined by unpaired *t*-test. Differentially expressed genes from RNA-seq are defined by a *p* < 0.01 and a fold change > ±1.5. Mean ± SEM. GSEA get set enrichment analysis, NES normalized enrichment score.

To unravel the mechanism behind the host protection mediated by Atg7 loss in IEC, we conducted a comprehensive analysis of the colonic gene expression profile in response to *C. rodentium* infection using RNA sequencing. Total RNA was extracted from the colons of infected WT and Atg7^ΔIEC^ mice at day 8 and day 18 p.i. A cohort of uninfected WT and *Atg7*^ΔIEC^ mice were also analyzed as controls. We first evaluated the impact of *C. rodentium* infection in both WT and *Atg7*^ΔIEC^ and then identified the ATG7-dependent gene expression program at day 8 and day 18 p.i. 1475 and 1401 genes displayed a fold change greater than 1.5 with a false discovery rate (FDR) below 0.01 in infected *Atg7*^ΔIEC^ mice respectively at day 8 and 18 p.i, among which 576 and 637 were also induced in control animals, respectively (Fig. 2a). Functional gene ontology analysis of these datasets using ClueGO revealed that the top ten biological pathways of the genes commonly upregulated in both WT and *Atg7*^ΔIEC^ mice at the peak of infection (8 days p.i) are associated with inflammation and infection (response to cytokine, defense response, response to other organisms). Additionally, these pathways include other functional categories previously linked to *C. rodentium* infection, such as the mitotic cell cycle and metabolic rewiring affecting cholesterol biogenesis (Fig. 2b). However, the ClueGO analysis of differentially expressed genes at day 8 p.i between WT and *Atg7*^ΔIEC^ mice underscored that cytokine production, immune and inflammatory responses were downregulated in ATG7-deficient mice. In contrast, a drug and hormone metabolic signature was induced upon ATG7 deficiency at day 8 p.i (Fig. 2c). Notably, the induction of several genes encoding pro-inflammatory interleukins, chemokines, and interferons was impaired in the colon of *Atg7*^ΔIEC^ mice at day 8 p.i (Fig. 2d). Similarly, Gene Set Enrichment Analysis (GSEA) revealed reduced expression of genes involved in tumor necrosis factor (TNFα), interferon-alpha (IFNα) and interferon-gamma (IFNγ) responses in ATG7-deficient colon at both day 8 and day 18 p.i (Fig. 2e–f). Additionally, these analyses revealed decreased expression of hallmarks of inflammatory response, including IL-6-JAK-Stat3 signaling, complement and IL-2-Stat5 signaling in *Atg7*^ΔIEC^ colons at day 8 p.i (Fig. 2e). Consistently, we confirmed by qRT-PCR the decreased mRNA levels of IL-1α, IL-1β, and IL-6 in the colon of *Atg7*^ΔIEC^ animals (Fig. 2g). Accordingly, the secretion of IL-1β, IL-6, and TNFα in colonic explant cultures was markedly reduced at day 8 p.i (Fig. 2h). At 18 p.i, a common immune response signature and an ATG7-dependent signature related to metabolism and morphogenesis were also identified (Fig. S3). Altogether, these analyses indicate that the loss of ATG7 impairs part of the inflammatory response during *C. rodentium* infection.

### IEC-specific *Atg7* deletion alters immune response during *C. rodentium* infection

**Fig. 3:**
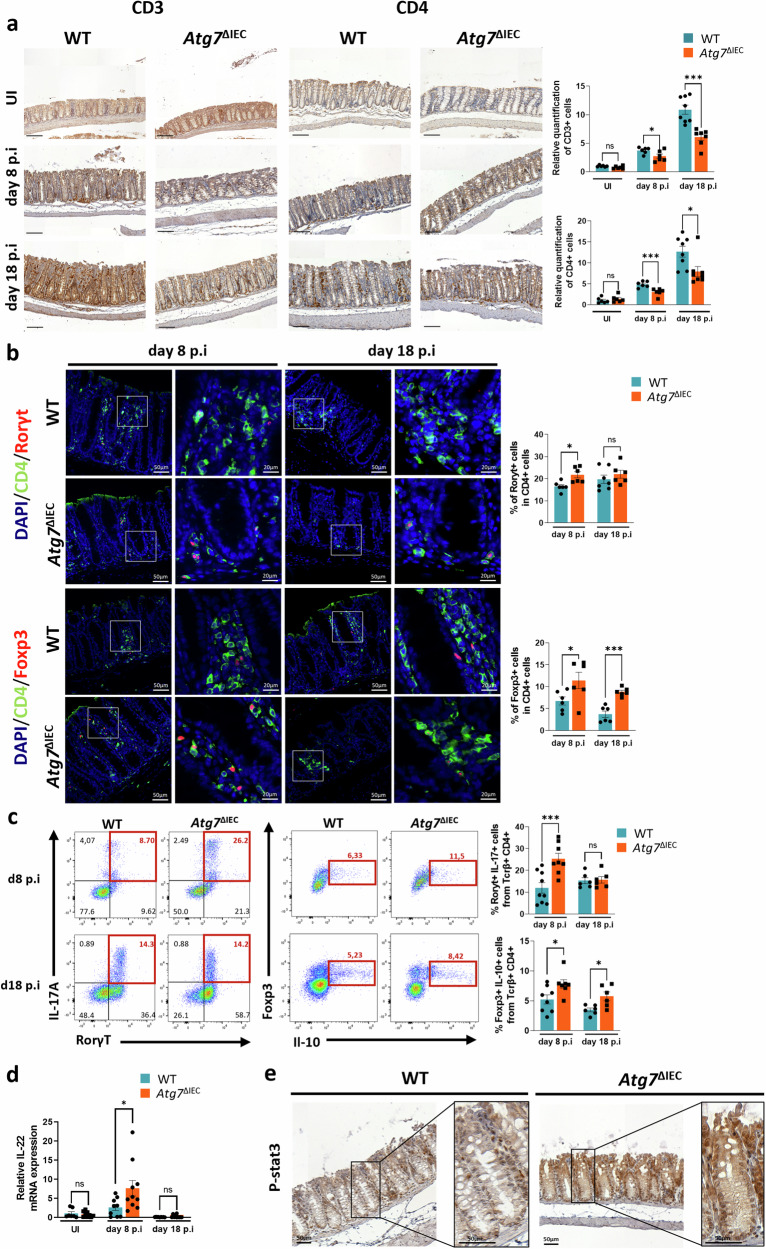
Loss of Atg7 modulates T lymphocytes recruitment and shapes Th17/Treg responses during *C. rodentium* infection. **a** Representative IHC staining for CD3 and CD4 on colonic sections from WT and *Atg7*^ΔIEC^ mice before and after infection at day 8 and day 18 p.i. Quantification of CD3-positive cells (UI: *n* = 6 WT mice, *n* = 6 *Atg7*^ΔIEC^ mice; at day 8 p.i: *n* = 6 WT mice, *n* = 6 *Atg7*^ΔIEC^ mice; at day 18 p.i: *n* = 8 WT mice and *n* = 7 *Atg7*^ΔIEC^ mice from two independent experiments) and CD4^+^ cells (UI: *n* = 6 WT mice, *n* = 6 *Atg7*^ΔIEC^ mice; at day 8 p.i: *n* = 6 WT mice, *n* = 6 *Atg7*^ΔIEC^ mice; at day 18 p.i: *n* = 8 WT mice and *n* = 7 *Atg7*^ΔIEC^ mice from two independent experiments). **b** Representative IF staining for CD4/Rorγt and CD4/Foxp3 on colonic sections from WT and *Atg7*^ΔIEC^ mice at day 8 and day 18 p.i. Quantification of CD4 and Rorγt double-positive cells (at day 8 p.i: *n* = 6 WT mice, *n* = 6 *Atg7*^ΔIEC^ mice; at day 18 p.i : *n* = 7 WT mice and *n* = 6 *Atg7*^ΔIEC^ mice from two independent experiments) and CD4 and Foxp3 double-positive cells (at day 8 p.i: *n* = 6 WT mice, *n* = 6 *Atg7*^ΔIEC^ mice and at day 18 p.i : *n* = 6 WT mice, *n* = 6 *Atg7*^ΔIEC^ mice from two independent experiments). **c** Fluorescence-activated cell sorting (FACS) analysis of T effector cells harvested from colonic lamina propria during *C. rodentium* infection. Representative flow cytometry plots of Rorγt^+^ IL-17A^+^ cells (Th17) and Foxp3^+^IL-10^+^ cells (Treg) within CD4^+^ Tcrβ^+^ cells from the colon of WT and *Atg7*^ΔIEC^ mice at day 8 and day 18 p.i. Quantification of Rorγt^+^ IL-17A^+^ cells (Th17) (at day 8: *n* = 9 WT mice, *n* = 8 *Atg7*^ΔIEC^ mice; at day 18 p.i: *n* = 6 WT mice, *n* = 6 *Atg7*^ΔIEC^ mice from two independent experiments) and Foxp3^+^IL-10^+^ cells (Treg) (at day 8: *n* = 8 WT mice, n = 7 *Atg7*^ΔIEC^ mice; at day 18 p.i: *n* = 6 WT mice, *n* = 6 *Atg7*^ΔIEC^ mice from two independent experiments) in the lamina propria. **d** Relative IL-22 mRNA levels assessed by qRT-PCR on colonic epithelium from UI, day 8 or day 18p.i from WT and *Atg7*^ΔIEC^ mice (UI: *n* = 7 WT mice, *n* = 9 *Atg7*^ΔIEC^ mice; at day 8 p.i: *n* = 9 WT mice, *n* = 10 *Atg7*^ΔIEC^ mice; at day 18 p.i: *n* = 9 WT mice and *n* = 10 *Atg7*^ΔIEC^ mice from two independent experiments). **e** Representative phospho-STAT3 staining of colonic sections from WT and *Atg7*^ΔIEC^ mice at day 8 p.i. Black scale bar =100 µm. Significant differences: *p < 0.05, **p < 0.01, ***p < 0.001, ns not significant, determined by unpaired *t*-test. Mean ± SEM.

Innate and adaptive immune responses serve as the first lines of defense against the invasion and expansion of enteric pathogens. To examine shifts in immune cell populations, which act as primary effectors in *C. rodentium* infection [23, 24], we quantified immune cells within the lamina propria across different stages of infection. In *Atg7*^ΔIEC^ mice, we observed an increase in inflammatory monocytes, macrophages, and neutrophils during the initial colonization phase at day 4 p.i. However, by day 8 p.i, these differences between WT and ATG7-deficient mice had largely resolved in the colonic lamina propria, as shown by flow cytometry (Fig. S4a–e). Notably, neutrophil percentages trended downward around day 8, paralleled by a reduction in infiltrating myeloperoxidase MPO^+^ neutrophils in the *Atg7*^ΔIEC^ colonic mucosa at this time point (Fig. S4f). This reduction correlated with the decreased expression of key chemokines for neutrophils (CCL2, CCL7) and chemokine receptor mainly expressed by neutrophils (CXCR2), which are prominent features of the colonic host response to *C. rodentium* (Fig. 2d). We observed a reduction in the recruitment of CD3^+^ and CD4^+^ T cells in the colon of *Atg7*^ΔIEC^ mice at day 8 and day 18 p.i compared to WT (Fig. 3a). However, there was an increase in the percentage of both T helper 17 cells (Th17), expressing both CD4 and RORγt, at day 8 p.i and in CD4^+^Foxp3^+^Tregs at day 8 and day 18 p.i in the colon of *Atg*7^ΔIEC^ mice, respectively (Fig. 3b). Consistently, profiling of immune cells in the lamina propria by flow cytometry confirmed an increased proportion of Th17 cells at day 8 p.i and in Treg at day 8 and day 18 p.i in the colon of *Atg7*^ΔIEC^ mice, respectively (Fig. 3c). Similarly, the mRNA levels of Th17-associated factors such as IL-17A and IL-17F were significantly higher in *Atg*7^ΔIEC^ than in WT colon at day 8 p.i (Fig. S4g). These data are consistent with the resistance to *C. rodentium* conferred by *Atg7* deletion, as Th17 cells play a pivotal role in host protection against *C. rodentium*, and Tregs are crucial for establishing a functional Th17 response while also limiting bacterial dissemination [25]. Furthermore, we observed higher colonic IL-22 expression at day 8 p.i in *Atg7*^ΔIEC^ mice compared to WT, associated with increased STAT3 phosphorylation—a recognized marker of mucosal wound healing (Fig. 3d, e) [27]. The secretion of IL-22 in colonic explant cultures from uninfected WT and *Atg7*^*ΔIEC*^ mice was similar (Fig. S4h), confirming that the induction of IL-22 observed on day 8 post infection in *Atg7*^*ΔIEC*^ mice is dependent on *C. rodentium* infection. Altogether, our results suggest that *Atg7* deletion may reduce tissue damage by enabling early pathogen control via mononuclear phagocytosis, followed by enhanced Treg and Th17 cell responses and activation of IL-22/Stat3 signaling pathways, thereby promoting immune protection and epithelial regeneration.

### ATG7-deficiency drive intestinal dysbiosis that is sufficient to confer resistance against *C. rodentium* infection

**Fig. 4:**
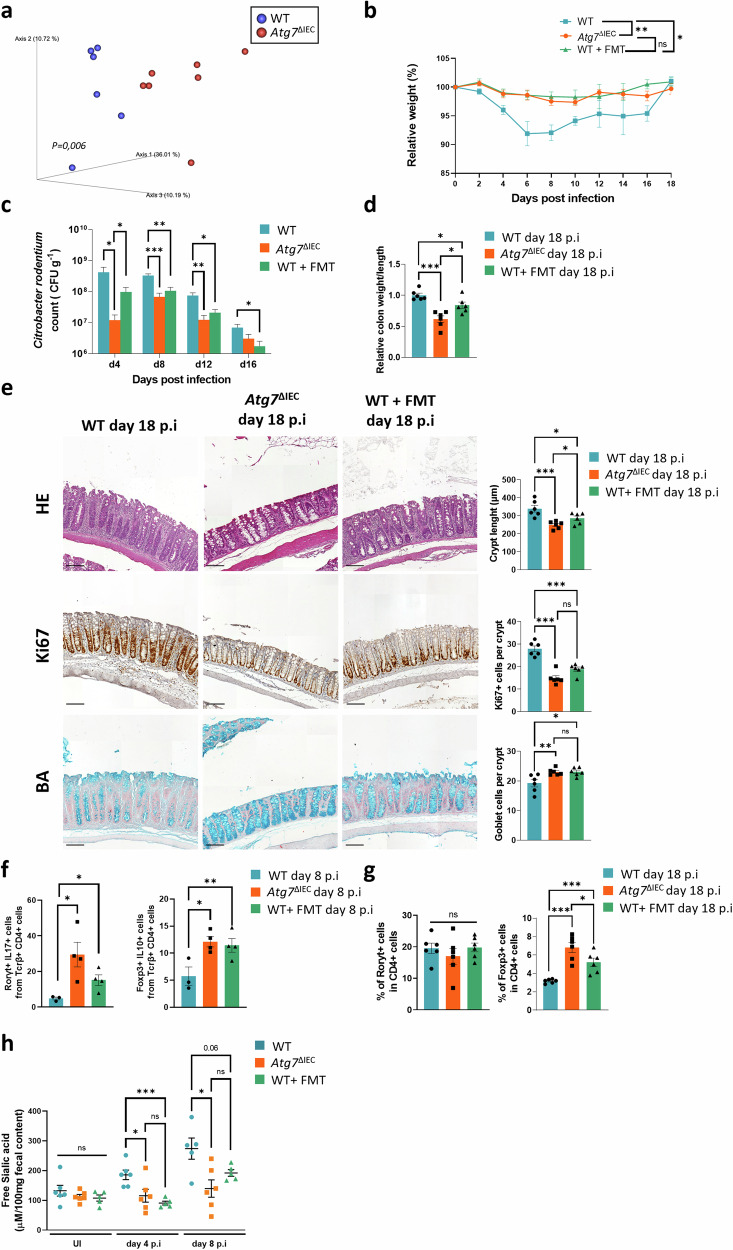
Microbiota transplantation of *Atg7* deficient mice protects WT mice from *C. rodentium* infection. **a** Principal component analysis of bacterial diversity of feces from WT and *Atg7*^ΔIEC^ mice (*n* = 6 WT mice, *n* = 6 *Atg7*^ΔIEC^ from two independent experiments, permanova *p*-value = 0.006), **b** Body weight changes after oral infection with *C. rodentium* of WT, *Atg7*^ΔIEC^ mice and WT mice with fecal transplantation from *Atg7*^ΔIEC^ mice (WT + FMT) (*n* = 6 mice per condition, from two independent experiments). Two-way ANOVA with Greenhouse-Geisser correction. **c** Quantification of bacterial burden in stool (CFU/g) over time from WT, *Atg7*^ΔIEC^ and WT + FMT mice (*n* = 6 mice per condition from two independent experiments). **d** Relative quantification of colon weight/length ratio from WT, *Atg7*^ΔIEC^ and WT + FMT mice at day 18 p.i (*n* = 6 mice per condition from two independent experiments). **e** Representative hematoxylin & eosin (H&E), alcian blue (BA), Ki67 staining on colonic sections from WT, *Atg7*^ΔIEC^ and WT + FMT mice at day 18 p.i. Quantification of colonic crypts length, Ki67 positive cells per crypts and BA-positive cells per crypt (*n* = 6 mice per condition from two independent experiments). **f** Percentage of Rorγt^+^ IL-17A^+^ cells (Th17) and Foxp3^+^IL-10^+^ cells (Treg) within the CD4^+^ cells obtained by FACS analysis from the lamina propria of the colon of WT, *Atg7*^ΔIEC^ and WT + FMT mice at day 8 p.i (*n* = 3 WT mice, *n* = 4 *Atg7*^ΔIEC^ mice, n = 4 *WT* + FMT mice). **g** Quantification by immunostainings of CD4 and Rorγt double-positive cells (*n* = 6 mice per condition from two independent experiments) and CD4 and Foxp3 double-positive cells (*n* = 6 mice per condition from two independent experiments) from the colon of WT, *Atg7*^*ΔIEC*^ and WT + FMT mice at day 18 p.i. **h** Levels of free sialic acid in feces from WT, *Atg7*^ΔIEC^ and WT + FMT mice before infection (UI) and after *C. rodentium* infection at day 4 p.i and 8 p.i (UI: *n* = 6 WT, *n* = 6 *Atg7*^ΔIEC^ *n* = 5 WT + FMT; day 4 p.i: *n* = 6 WT mice, *n* = 6 *Atg7*^ΔIEC^ mice, *n* = 5 WT + FMT; day 8 p.i: *n* = 5 WT mice, *n* = 6 *Atg7*^ΔIEC^ mice, *n* = 5 WT + FMT mice). Black scale bar = 100 µm. Mean ± SEM. Significant differences: *p < 0.05, **p < 0.01, ***p < 0.001, ns not significant, determined by unpaired *t*-test.

The composition of the intestinal microbiota has been recognized as a central factor influencing the immune response and susceptibility to *C. rodentium* infection [16]. We thus investigated intestinal microbiota composition through Illumina-based sequencing of the 16S rRNA gene (V3-V4 region). Principal component analysis of the Jaccard matrix distance revealed a significant alteration in the overall composition of fecal microbiota between WT and *Atg7*^ΔIEC^ mice (Figs. 4a and S5). Next, in order to investigate to which extent the observed differences in intestinal microbiota between WT and Atg7^ΔIEC^ mice are playing a role in dictating infection outcome, we employed fecal microbiota transplantation (FMT). WT mice underwent one week of antibiotic treatment and were then gavaged with stool harvested from *Atg7*^ΔIEC^ mice prior to *C. rodentium* infection hereafter referred to as WT + FMT. WT + FMT mice exhibited alleviated body weight loss during the infection compared to infected WT animals (Fig. 4b). No significant difference in body weight was observed throughout the infection in *Atg7*^ΔIEC^ and WT + FMT mice Additionally, WT + FMT animals reported reduced pathogen load throughout the infection and a diminished colon weight/length ratio at day 18 p.i (Fig. 4c, d). Histological analysis confirmed the beneficial effects of FMT with clinical outcome. WT + FMT mice displayed limited crypt hyperplasia, proliferation, and goblet cell loss compared to WT colon although the crypt length remained longer in WT + FMT compared to *Atg7*^ΔIEC^ mice (Fig. 4e). CD3 and CD4 staining indicated a reduced number of lymphocytes in the colon of WT + FMT mice compared to WT animals but remained higher than in *Atg7*^ΔIEC^ mice (Fig. S6a). Immunofluorescence and FACS analyses revealed an increased proportion of Th17 cells at day 8 p.i and in Treg at day 8 and day 18 p.i in the colon of WT + FMT mice compared to WT (Figs. 4f–g and S6b). Although the number of Th17 and Treg cells was higher in the colon of *Atg7*^ΔIEC^ than in the WT + FMT model at day 8 and day 18 p.i, respectively, our data underscore the significance of the microbiota in the effects of ATG7 deficiency in IECs on resistance to *C. rodentium* infection.

During infection, luminal *C. rodentium* must cross the intestinal mucus layer before reaching the underlying intestinal epithelium. Sialic acids are monosaccharides generally found at the end of mucins. Many commensals and pathogens use sialic acids as an energy source to survive in mucus-covered host environments, such as the gut [28]. A recent study revealed that *C. rodentium* employs sialic acid to promote its expansion and colonization of the intestinal epithelium [29]. However, for *C. rodentium* to access sialic acid, other bacteria must first release it from the mucus. Thus, the amount of Free Sialic Acid (FSA) in feces can predict the susceptibility of mice to *C. rodentium*. We measured fecal FSA levels in control, *Atg7*^ΔIEC^, and WT + FMT mice, both with and without *C. rodentium* infection (Fig. 4h). Prior to infection, FSA levels were similar across control, *Atg7*^ΔIEC^, and WT + FMT groups. Following infection, FSA levels were increased in control mice at days 4 and 8 p.i, compared to *Atg7*^*ΔIEC*^ mice. Moreover, control mice transplanted with microbiota from *Atg7*^*ΔIEC*^ mice showed a reduced FSA level compared to infected non-transplanted control mice, confirming a diminished impact of infection in this context. These results suggest that, upon infection with *C. rodentium*, the microbiota of autophagy-deficient mice exhibits less sialidase activity, releasing less FSA, which may contribute to limiting *C. rodentium* infection.

### Resistance to *C. rodentium* mediated by *Atg7* deficiency is dependent on Gram-positive bacteria

**Fig. 5:**
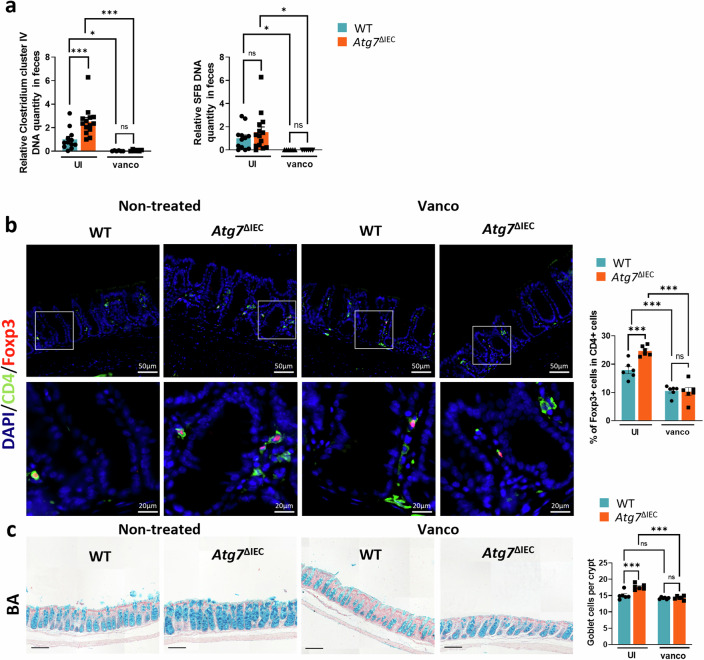
Treg and goblet cell induction conferred by Atg7 inhibition is abolished by vancomycin treatment. **a** Impact of vancomycin treatment on the abundance of *Clostridium* cluster IV (*C. leptum group)* and Segmented Filamentous Bacteria (SFB) in WT and *Atg7*^ΔIEC^ mice. Bacterial DNA was extracted from feces (*n* = 12 WT mice, *n* = 14 *Atg7*^ΔIEC^ mice, *n* = 6 WT+vanco mice, *n* = 6 Atg7^ΔIEC^+vanco mice). **b** Representative IHC stainings for Foxp3 and CD4 on colonic sections from WT and *Atg7*^ΔIEC^ mice non-treated and treated with vancomycin. Quantification of CD4 and Foxp3 double-positive cells in the lamina propria (*n* = 6 mice per condition from two independent experiments). **c** Representative alcian blue (BA) staining on colonic sections from WT and *Atg7*^ΔIEC^ mice non-treated and treated with vancomycin. Quantification of BA-positive cells per crypt (*n* = 6 mice per condition from two independent experiments). Black scale bar = 100 µm. Mean ± SEM. Significant differences: *p < 0.05, **p < 0.01, ***p < 0.001, ns not significant, determined by unpaired *t*-test.

**Fig. 6:**
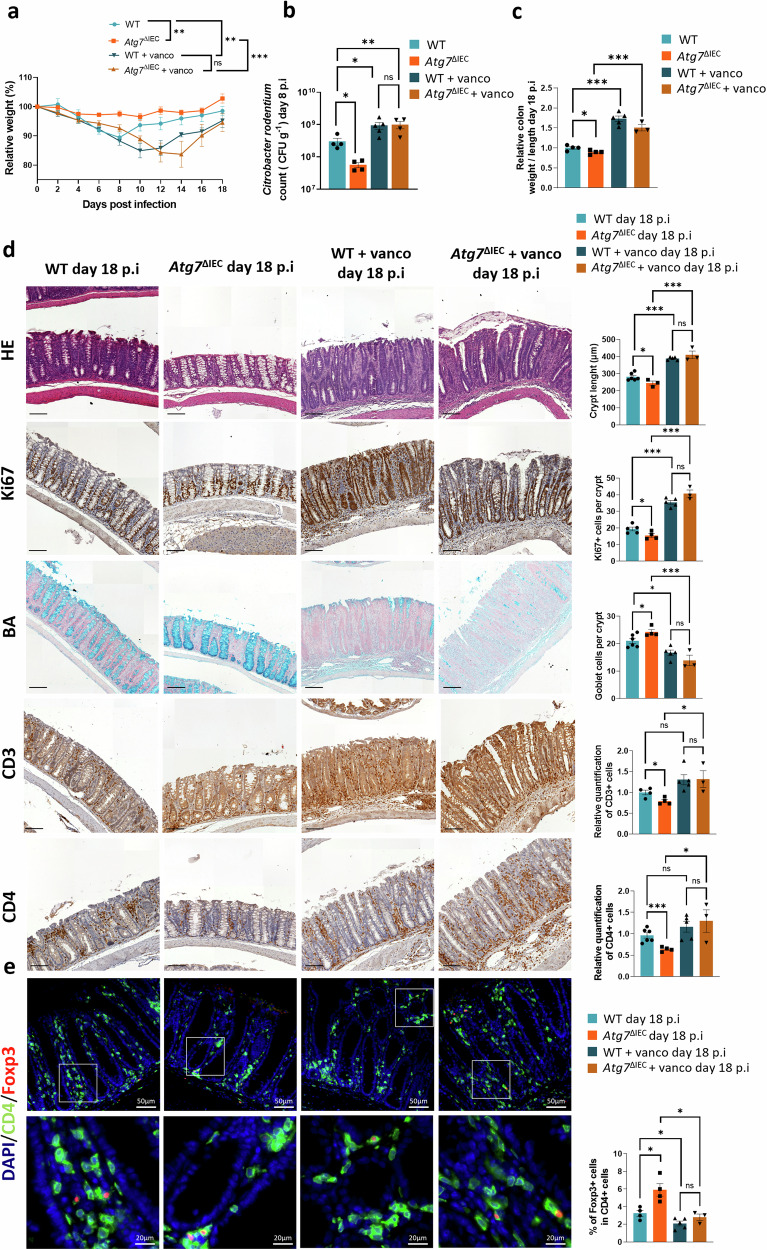
Vancomycin treatment alters resistance to *C. rodentium* in Atg7 mutant and WT mice. **a** Body weight changes of WT and *Atg7*^ΔIEC^ mice infected with *C. rodentium* after vancomycin treatment (*n* = 5 WT mice, *n* = 4 *Atg7*^ΔIEC^ mice, *n* = 4 WT+vanco mice, *n* = 3 *Atg7*^ΔIEC^+vanco mice). Two-way ANOVA with Greenhouse-Geisser correction. **b** Quantification of bacterial burden in stool (CFU/g) at day 8 after *C. rodentium* infection from WT and *Atg7*^ΔIEC^ mice non-treated and vancomycin-treated (*n* = 4 WT, *n* = 4 *Atg7*^ΔIEC^, *n* = 5 WT + vanco, *n* = 4 *Atg7*^ΔIEC^ + vanco). **c** Relative quantification of colon weight/length ratio from WT and *Atg7*^ΔIEC^ mice non-treated and vancomycin-treated at day 18 p.i (*n* = 4 WT mice, *n* = 4 *Atg7*^ΔIEC^ mice, *n* = 5 WT + vanco mice, *n* = 3 *Atg7*^ΔIEC^+vanco mice). **d** Representative hematoxylin & eosin (H&E), Ki67, alcian blue (BA), CD3, and CD4 staining of colonic sections from WT and *Atg7*^ΔIEC^ mice non-treated and vancomycin-treated at day 18 p.i. Quantification of colonic crypts length (*n* = 6 WT mice, *n* = 3 *Atg7*^ΔIEC^ mice, *n* = 5 WT + vanco mice and *n* = 3 *Atg7*^ΔIEC^+vanco mice). Quantification of Ki67 positive cells per crypts (*n* = 5 WT mice, *n* = 4 *Atg7*^ΔIEC^ mice, *n* = 5 WT + vanco mice and *n* = 3 *Atg7*^ΔIEC^ + vanco mice). Quantification of BA-positive cells per colonic section (*n* = 5 WT mice, *n* = 4 *Atg7*^ΔIEC^ mice, *n* = 5 WT + vanco mice and *n* = 3 *Atg7*^ΔIEC^ + vanco mice). Quantification of CD3 positive cells per crypt (*n* = 4 WT mice, *n* = 4 *Atg7*^ΔIEC^ mice, *n* = 5 WT + vanco mice and *n* = 3 *Atg7*^ΔIEC^ + vanco mice). Quantification of CD4 positive cells per colonic section (*n* = 6 WT mice, *n* = 4 *Atg7*^ΔIEC^ mice, *n* = 5 WT+vanco mice, *n* = 3 *Atg7*^ΔIEC^ + vanco mice). **e** Representative IHC staining for CD4 and Foxp3 on colonic sections from non-treated and vancomycin-treated WT and *Atg7*^ΔIEC^ mice at day 18 p.i. Quantification of CD4 and Foxp3 double-positive cells per colonic sections (*n* = 4 WT mice, *n* = 4 *Atg7*^ΔIEC^ mice, *n* = 5 WT+vanco differences: *p < 0.05, **p < 0.01, ***p < 0.001, ns not significant, determined by unpaired *t*-test.

The obtained results from WT-FMT mice prompted us to investigate the presence of specific bacterial species known to confer protection against *C. rodentium* infection in Atg7^ΔIEC^ mice prior to *C. rodentium* infection. For this purpose, we used qRT-PCR approach in order to bring semi-quantitative information. We observed no major differences in the relative abundance of Gram-negative bacteria, including *Verrucomicrobia*, *Proteobacteria* (*classes α and δ*), and *E. coli*, and Bacteroides in the feces of *Atg7*^*ΔIEC*^ mice compared to WT mice (Fig. S7). Given the immune response observed, we also examined the presence of Gram-positive bacteria Segmented Filamentous Bacteria (SFB), known potent inducers of Th17 cells, and *Clostridia* cluster IV, recognized for inducing Treg cells [30, 31]. Such an approach revealed a higher colonization of *Clostridium* cluster IV in the feces of *Atg7*^ΔIEC^ mice compared to WT mice and an induction of Treg in the lamina propria of *Atg*7^ΔIEC^ mice (Fig. 5a). No difference in SFB was observed between the two genotypes (Fig. 5a). To further delineate the role of commensal bacteria and in particular *Clostridium* cluster IV present in *Atg7*^ΔIEC^ mucosa prior *C. rodentium* infection, we treated them with vancomycin, which preferentially target Gram-positive bacteria. As expected, vancomycin treatment eliminated *Clostridium* clusters IV and SFB bacteria both in *Atg7*^ΔIEC^ and WT mice (Fig. 5a). Compared to their respective controls, both *Atg7*^ΔIEC^ and WT mice treated with vancomycin exhibited significantly fewer Tregs in the colon, confirming the dominant role for Gram-positive bacteria in Treg accumulation (Fig. 5b). Moreover, vancomycin treatment in *Atg7*^ΔIEC^ mice abolished the induction of goblet cells observed in untreated mice, suggesting that alterations of the microbiota due to ATG7 deficiency, particularly Gram-positive bacteria, contribute to an increased number of goblet cells (Fig. 5c). We then investigated the impact of vancomycin treatment on the clearance and resistance to *C. rodentium* infection observed in *Atg7*^ΔIEC^ mice. Treatment of *Atg7*^ΔIEC^ mice with vancomycin for 13 days prior to *C. rodentium* infection resulted in severe weight loss throughout the infection and increased bacterial shedding compared to their untreated counterparts (Fig. 6a, b). Accordingly, vancomycin-treated *Atg*7^ΔIEC^ mice took longer to clear the infection, as evidenced by bioluminescence imaging showing a higher *C. rodentium* signal in these mice (Fig. S1b). Morphometric analysis of the colon revealed that *Atg7*^ΔIEC^-infected mice treated with vancomycin exhibited a higher colon weight/length ratio at day 18 p.i (Fig. 6c). Our results also indicate that vancomycin-treated WT mice could not clear *C. rodentium* (Fig. 6a–c). Thus, our data suggest that vancomycin treatment reverses the difference in host susceptibility of *Atg7*^ΔIEC^ but also sensitizes WT mice to *C. rodentium* infection. Histological analyses at day 18 p.i revealed strong hyperplasia associated with a drastic increase in epithelial cell proliferation and goblet cell loss in vancomycin-treated WT and *Atg7*^ΔIEC^ mice compared to untreated mice (Fig. 6d). Vancomycin treatment restored CD3 and CD4 infiltration in *Atg7*^ΔIEC^ mice and reduced the frequency of Tregs compared to untreated *Atg7*^ΔIEC^ mice at day 18 p.i (Fig. 6e). Altogether these results suggest that the Gram-positive microbiota is crucial for the host to eliminate *C. rodentium* infection, and *Atg7* deficiency affects the abundance of Gram-positive bacteria, contributing to resistance to *C. rodentium* infection.

## Discussion

In this study, we report that the specific loss of Atg7 in IEC provides protection against *C. rodentium* infection by reducing the colonic burden of the pathogen. This protective effect is associated with a reduction in intestinal pathology, including decreased epithelial hyperplasia, preservation of goblet cells, and alleviation of mucosal inflammation induced by the pathogen (Fig. S8). At the initial phase of infection, an increase in neutrophils along with monocytes and macrophages with no change in dendritic cells has been observed in the absence of ATG7. Interestingly, such increase in neutrophils reversed in ATG7 deficient mice at a later time with concomitantly reduced expression of pro-inflammatory cytokines, enhancement of Treg and Th17 cells with reduced bacterial burden. Even though, these results imply the role for early phase inflammatory cell type recruitment in ATG7 deficient mice, it has to be still coupled to late phase increased protective immune cells type (Th17 and Treg) in conferring resistance against *C. rodentium*. However, the mechanistic details regulating such time lapse change in immune cells population during pathogen infection in ATG7 deficient mice remain to be explored yet. Furthermore, our data also underscore the pivotal role of the microbiota in the resistance conferred by Atg7 deletion against *C. rodentium*, with a particular emphasis on Gram-positive bacteria. Notably, these bacteria can be effectively transferred through FMT.

The impact of autophagy deficiency on *C. rodentium* infection has been investigated in various genetic mouse models, but a consensus on the infection outcome has not been reached. Furthermore, mechanistic insights that might reconcile these discrepancies have not yet been explored. While deletion of *Atg16L1* in IEC has been reported to have no impact on *C. rodentium* infection, deletion of *Atg7* in IEC has been shown to confer susceptibility [17, 32]. However, our findings, along with other reports, demonstrate that mutations or absence of *Atg7*, *Atg4b*, *LC3b*, or *Atg16L1* can trigger a protective response against the infection [18, 19]. The discrepancies between the findings do not seem to be linked to the specific Atg genes targeted. Instead, they may be directly influenced by the particular microbiome, which could act as a confounding factor contributing to the inconsistent data. It is well acknowledged that inhibition of autophagy cause dysbiosis but in addition to host genetic factors, diet and the host environment exert dominant influences on microbiota composition and consequently can impact differently the disease susceptibility [33]. Accordingly, C57BL/6N mouse lines from different breeding facilities display unique microbiota profiles ultimately influencing their susceptibility to *C. rodentium* infection [34]. The variation observed in different studies utilizing autophagy-deficient mice may also be attributed to the health status of the mice, particularly the presence of enteric viruses such as norovirus. These viruses have been shown to impact intestinal homeostasis and mucosal immunity similarly to commensal bacteria [35]. The mechanism mediated by autophagy involved in the resistance to *C. rodentium* has been characterized in Atg16L1 hypomorphic (Atg16L1HM) mice. Accordingly, to our study, the gut microbiota has been shown to mediate the resistance to *C. rodentium* of Atg16L1HM mice [19]. However, the benefit conferred by Atg16L1 mutation is dependent on microbiota-triggered IFN-I response, which is absent in infected Atg7^ΔIEC^ mice. This distinct response may be the consequence of the varying compositions of specific microbes between the two mouse colonies.

Mounting evidences suggest that dysregulated autophagy mediates dysbiosis. Indeed, we and others have previously shown Paneth cell defects linked to impaired autophagy. We previously demonstrated that Paneth cells from *Atg7*^ΔIEC^ mice produce reduced amounts of antimicrobial peptides and exhibit both abnormal lysozyme distribution and reduced granule numbers [2, 7]. These findings align with observations made by Cadwell and colleagues in mice deficient in Atg5, Atg16L1, and Atg7 [10, 36]. Such defects impair the secretion antimicrobial substances, leading to functional consequences on the composition and abundance of indigenous microbes within the intestinal lumen [37]. Additionally, our data confirm that autophagy regulates mucus secretion from colonic goblet cells [21, 22]. Notably, the loss of autophagy proteins disrupts the function of various secretory cell types, not only in the intestine but also in vasculature, osteoclasts, and immune cells, although the exact mechanisms remain unclear [38]. Nevertheless, our results are consistent with a model in which the dysbiosis observed in Atg7 mice limits pathogen access and promotes an efficient immune protective response. Our findings imply that the imbalance in the microbiota resulting from Atg7 deletion may provide protection against *C. rodentium* infection by limiting pathogen access to IEC through reduced level of sialic acid. We found similar levels in uninfected WT and *Atg7*^*ΔIEC*^ mice. However, following *C. rodentium* infection, FSA levels increased in WT mice. Interestingly, *C. rodentium* itself does not appear to express sialidases directly [39]. The observed rise in FSA in WT mice could be attributed to increased bacterial sialidase activity, likely driven by shifts in gut microbiota composition during infection. The differing FSA levels between WT and *Atg7*^*ΔIEC*^ mice post-infection may reflect underlying differences in microbiota composition between the two genotypes, even after *C. rodentium* colonization. Future studies utilizing shotgun metagenomic analysis on a larger cohort of animals are essential to further elucidate the role of *Atg7* in microbiota composition and regulation. This approach will also enable the identification of bacterial species with sialidase activity, helping to pinpoint those that contribute to *C. rodentium* susceptibility.

Finally, our findings highlight that microbiota-dependent protection against *C. rodentium* is associated with an increased proportion of Th17 cells at the peak of the infection and a sustained elevation of Treg cells throughout the infection. The composition of the microbiota is known to impact T cell differentiation, including Th1, Th2, Th17, and Treg cells [40–42]. Specifically, within the Gram-positive bacteria, we observed a rise in *Clostridium* cluster IV, a strain of clostridia known to promote the expansion and differentiation of Treg cells [34]. Importantly, while microbiota composition can vary significantly due to many environmental factors, our observations of heightened Clostridia abundance in Atg7-deficient mice are consistent with other gut microbiome analysis of Atg7 and Atg5 mutant mice [13, 14]. The underlying mechanism linking autophagy inhibition to elevated Clostridium colonization, particularly given their obligate anaerobic nature, remains unclear. However, this dysbiosis might be related to changes in intestinal oxygen levels, as supported by research indicating that intestinal oxygenation can directly impact microbial ecology [43]. Further investigation is needed to elucidate how autophagy might influence intestinal oxygenation. Treg cells have been demonstrated to play a pivotal role in protecting against *C. rodentium*. Depletion of Treg cells lead to compromised bacterial clearance, heightened body weight loss, and increased systemic dissemination of bacteria [25]. Furthermore, the presence of Treg cells reduces IL-2/STAT5 signaling and fosters Th17 differentiation. Accordingly, our findings suggest that IL-2/STAT5 signaling is part of the predominant downregulated canonical pathways in Atg7-deficient mice during the peak of *C. rodentium* infection. Based on these results, we propose that alterations in the microbiota of Atg7-deficient mice may mitigate inflammation triggered by *C. rodentium* infection by promoting a protective immune response through a balanced Th17/Treg immune response.

To conclude, our study emphasizes the influence of microbiota associated with autophagy dysfunction on susceptibility to *C. rodentium* and suggest the potential for leveraging the protective capacity of the intestinal microbiota, thereby presenting an exciting opportunity for the treatment of enteropathogenic infections.

## Supplementary information

Supplemental Table 1

Supplementary Figures

## Data Availability

The RNA sequencing data that support the finding of this study are available in the Gene Expression Omnibus with the accession number GSE279997. Unprocessed microbiota sequencing data are deposited in the European Nucleotide Archive PRJEB81558.
