# Supplementary material for: Inhibition of Atg7 in intestinal epithelial cells drives resistance against Citrobacter rodentium
Source: Cell Death Dis. 2025 Feb 19;16(1):112. doi: 10.1038/s41419-025-07422-5 (PMC11840101; doi:10.1038/s41419-025-07422-5)
Supplement: Supplementary file 1 — Supplemental Table 1 [file 41419_2025_7422_MOESM1_ESM.docx]

**Supplementary Table 1**

| **Target** | **Forward primer** | **Reverse primer** |
| --- | --- | --- |
| *Clostridium* cluster IV | CCTTCCGTGCCGSAGTTA | GAATTAAACCACATACTCCACTGCTT |
| *Clostridium* cluster XIVa | AAATGACGGTACCTGACTAA | CTTTGAGTTTCATTCTTGCGAA |
| *Genus Bacteroïdes* | GATCCAGCAATTCTGTGTGC | CGAATTTCACCTCTACACTTGT |
| Phylum *Verrucomicrobia* | GAATTCTCGGTGTAGCA | GGCATTGTAGTACGTGTGCA |
| Phylum *Fusobacteria* | GATCCAGCAATTCTGTGTGC | CGAATTTCACCTCTACACTTGT |
| Class *β- and γ-proteobacteria* | GTATAATGGGTCAGCGAC | CAGCATTCGCACTTCTGA |
| Class *δ-proteobacteria* | GGTGTAGGAGTGAARTCCGT | TACGTGTGTAGCCCTRGRC |
| *Escherichia coli* | CATGCCGCGTGTATGAAGA | CGGGTAACGTCAATGAGCAAA |
| SFB | GACGCTGAGGCATGAGAGCAT | GACGGCACGGATTGTTATTCA |
| 16S | GTGYCAGCMGCCGCGGTAA | GGACTACNVGGGTWTCTAAT |
| 18S | GTAACCCGTTGAACCCCATT | CCATCCAATCGGTAGTAGCG |
| IL-1α | GCTGAGCTTTGAGGGATGATC | GAGCGCTCACGAACAGTTG |
| IL-1β | CAACCAACAAGTGATATTCTCC | GATCCACACTCTCCAGCTGCA |
| IL-6 | TGGTCTGTTGTGGGTGGTATCC | CTTCCAGCCAGTTGCCTTCTTG |
| IL-17A | GGACTCTCCACCGCAATGA | GGCACTGAGCTTCCCAGATC |
| IL-17F | CCCCATGGGATTACAACATCAC | CATTGATGCAGCCTGAGTGTCT |
| IL-22 | CGCTGCCCGTCAACACCCGG | CTGATCTTTAGCACTGACTCCTCG |
