# Supplementary material for: Inhibition of Atg7 in intestinal epithelial cells drives resistance against Citrobacter rodentium
Source: Cell Death Dis. 2025 Feb 19;16(1):112. doi: 10.1038/s41419-025-07422-5 (PMC11840101; doi:10.1038/s41419-025-07422-5)
Supplement: Supplementary file 2 — Supplementary Figures [file 41419_2025_7422_MOESM2_ESM.pdf]

Figure S1

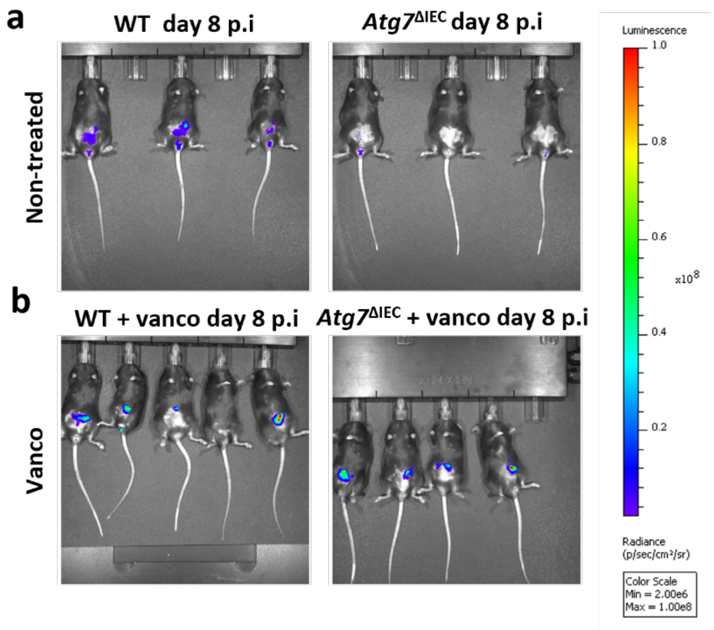

Fig S1: Effect of *Atg7* deletion on in *vivo* growth of *C. rodentium.* (a) *In vivo* bioluminescent imaging (BLI) of *C. rodentium* in representative WT and *Atg7*$^{\Delta IEC}$ mice at day 8 p.i. **(b)** *In vivo* BLI of *C. rodentium* in representative WT and *Atg7*$^{\Delta IEC}$ mice at day 8 p.i treated with vancomycin. The scale bar indicates signal intensity (photons s^{-1} cm^{-2} sr^{-1}).

Figure S2

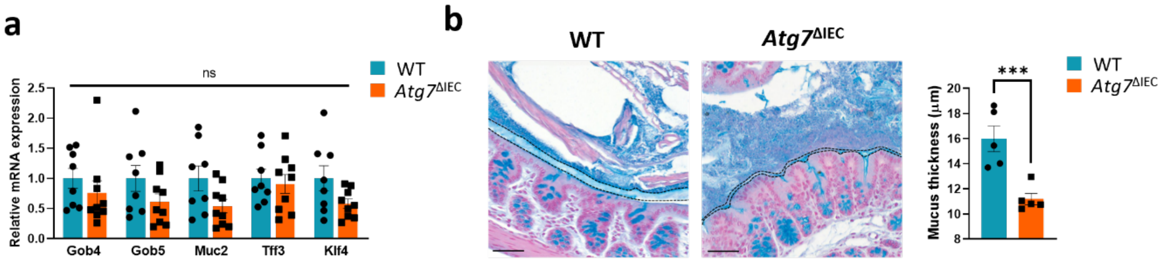

Fig S2: Effect of *Atg7* deletion on mucus (a) Relative Gob4, Gob5, Muc2, Tff3 and Klf4 mRNA level assessed by qRT-PCR on colonic epithelium from WT and *Atg7*$^{\Delta IEC}$ mice uninfected (n=8 WT; n=9 *Atg7*$^{\Delta IEC}$, two independent experiment). **(b)** Representative image of the inner mucus layer (delimited by the black dashed line) in proximal colon stained with alcian blue of WT and *Atg7*$^{\Delta IEC}$ mice. scale bar = 50µm. Quantification of the inner mucus layer thickness in n=5 WT and n=5 *Atg7*$^{\Delta IEC}$ mice. Significant difference: * p<0.05, ***p<0.001, ns = not significant, determined by unpaired *t*-test. Mean ± SEM.

Figure S3

day 18 p.i. vs UI: ClueGo analysis of common genes upregulated in WT and *Atg7*$^{\Delta IEC}$

GO ID	GO term	*P Value*
GO:0006952	defense response	0,00E+00
GO:0043207	response to external biotic stimulus	0,00E+00
GO:0051707	response to other organism	0,00E+00
GO:0098542	defense response to other organism	1,15E-42
GO:0034097	response to cytokine	1,39E-38
GO:0045087	innate immune response	3,22E-38
GO:0050776	regulation of immune response	3,96E-33
GO:0002682	regulation of immune system process	2,09E-32
GO:0071345	cellular response to cytokine stimulus	4,10E-32
GO:0031347	regulation of defense response	1,98E-27

day 18: ClueGo analysis of genes downregulated in *Atg7*$^{\Delta IEC}$ vs WT

GO ID	GO term	*P Value*
GO:0048518	positive regulation of biological process	3,46E-28
GO:1901564	organonitrogen compound metabolic process	6,91E-27
GO:0070887	cellular response to chemical stimulus	2,81E-25
GO:0048522	positive regulation of cellular process	9,43E-25
GO:0006810	transport	8,29E-24
GO:0006793	phosphorus metabolic process	3,17E-23
GO:0006796	phosphate-containing compound metabolic proc	6,47E-23
GO:0071310	cellular response to organic substance	4,68E-22
GO:0010033	response to organic substance	1,07E-21
GO:0071702	organic substance transport	6,80E-21

day 18: ClueGo analysis of genes upregulated in *Atg7*$^{\Delta IEC}$ vs WT

GO ID	GO term	*P Value*
GO:0007275	multicellular organism development	0,00E+00
GO:0009653	anatomical structure morphogenesis	0,00E+00
GO:0016043	cellular component organization	0,00E+00
GO:0035239	tube morphogenesis	0,00E+00
GO:0035295	tube development	0,00E+00
GO:0048731	system development	0,00E+00
GO:0072359	circulatory system development	0,00E+00
GO:0007399	nervous system development	2,80E-45
GO:0048522	positive regulation of cellular process	5,61E-45
GO:0004930	G protein-coupled receptor activity	4,76E-44

Fig S3: Colonic transcriptional response associated with *Atg7* deletion at day 18 after *C. rodentium* infection. ClueGO analysis of the top ten biological pathways of the genes upregulated at day 18 p.i both in WT and *Atg7*$^{\Delta IEC}$ colon compared to uninfected samples of the same genotype. ClueGO analysis of the top ten biological pathways of the up and down - regulated genes in *Atg7*$^{\Delta IEC}$ colon compared to WT at day 18 p.i. Differentially expressed genes from RNA-seq are defined by a p-value<0.01 and a fold change>±1.5.

Figure S4

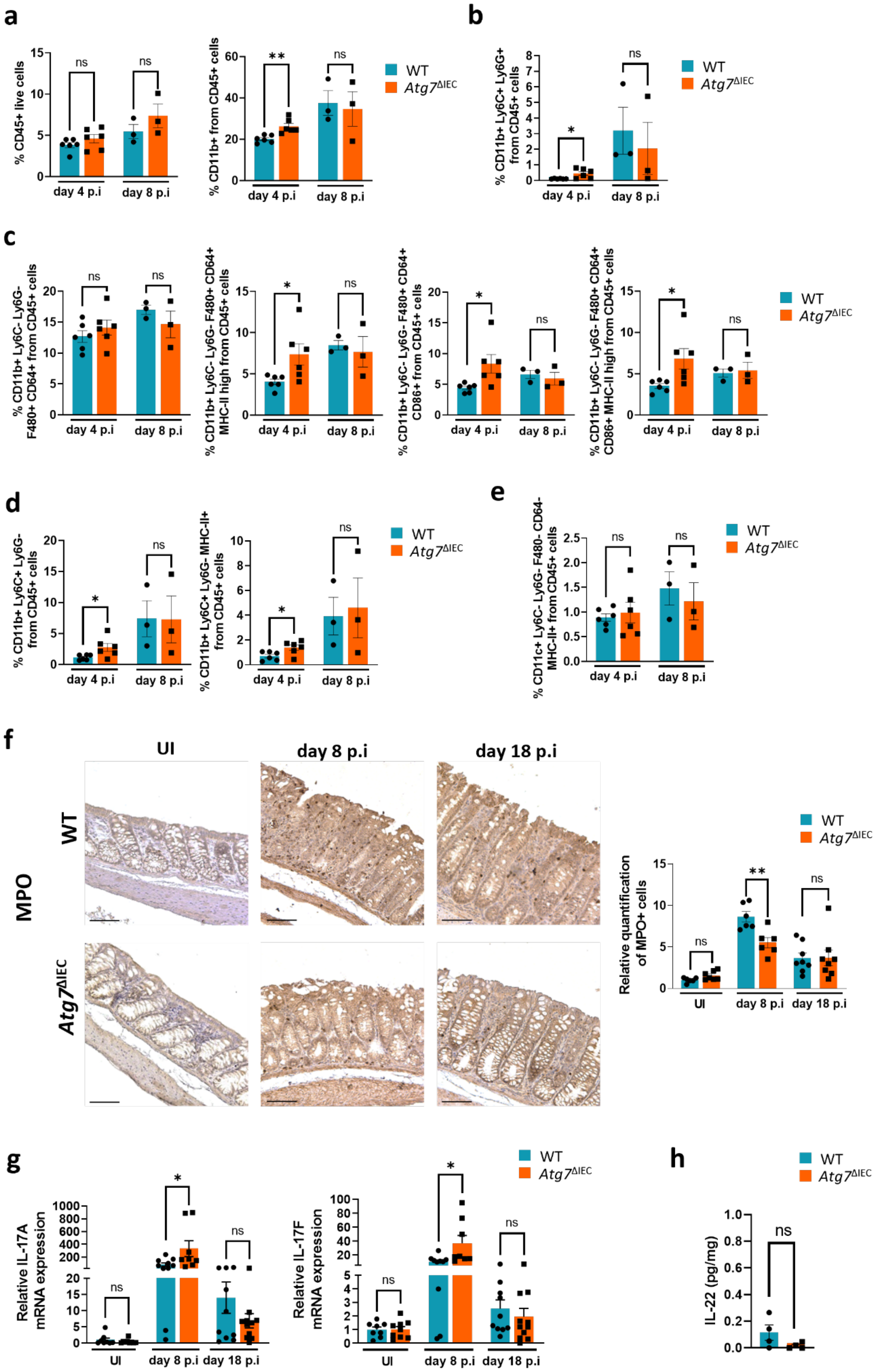

a
% CD45+ live cells
ns
ns
day 4 p.i
day 8 p.i
% CD11b+ from CD45+ cells
**
ns
WT
Atg7ΔIEC
b
% CD11b+ Ly6C+ Ly6G+ from CD45+ cells
*
ns
c
% CD11b+ Ly6C- Ly6G- F480+ CD64+ from CD45+ cells
% CD11b+ Ly6C- Ly6G- F480+ CD64+ MHC-II high from CD45+ cells
% CD11b+ Ly6C- Ly6G- F480+ CD64+ CD86+ from CD45+ cells
% CD11b+ Ly6C- Ly6G- F480+ CD64+ CD86+ MHC-II high from CD45+ cells
d
% CD11b+ Ly6C+ Ly6G- from CD45+ cells
% CD11b+ Ly6C+ Ly6G- MHC-II+ from CD45+ cells
e
% CD11c+ Ly6C- Ly6G- F480- CD64- MHC-II+ from CD45+ cells
f
UI
day 8 p.i
day 18 p.i
MPO
WT
Atg7ΔIEC
Relative quantification of MPO+ cells
g
Relative IL-17A mRNA expression
Relative IL-17F mRNA expression
h
IL-22 (pg/mg)

Fig S4: Effect of *Atg7* deletion on immune cell response after *C. rodentium* infection. **(a-e)** Fluorescence-activated cell sorting (FACS) analysis of innate immune cells harvested from colonic lamina propria of WT and *Atg7*$^{\Delta IEC}$ mice at day 4 and day 8 after *Citrobacter rodentium* infection. (at day 4 p.i: n=6 WT mice, n=6 *Atg7*$^{\Delta IEC}$ mice; at day 8 p.i: n=3 WT mice, n=3 *Atg7*$^{\Delta IEC}$ mice) **(a)** Quantification of CD45$^+$ and CD11b$^+$ cells among CD45$^+$ cells. **(b)** Quantification of CD11b$^+$ Ly6C$^+$ Ly6G$^+$ (neutrophils) within CD45$^+$ cells. **(c)** Quantification of CD11b$^+$ Ly6C$^-$ Ly6G$^-$ F480$^+$ CD64$^+$ cells, CD11b$^+$ Ly6C$^-$ Ly6G$^-$ F480$^+$ CD64$^+$ MHC-II^{high} cells, CD11b$^+$ Ly6C$^-$ Ly6G$^-$ F480$^+$ CD64$^+$ CD86$^+$ cells and CD11b$^+$ Ly6C$^-$ Ly6G$^-$ F480$^+$ CD64$^+$ CD86$^+$ MHC-II^{high} cells (macrophage cell types) within CD45$^+$ cells. **(d)** Quantification of CD11b$^+$ Ly6C$^+$ Ly6G$^-$ cells and CD11b$^+$ Ly6C$^+$ Ly6G$^-$ MHC-II$^+$ within CD45$^+$ cells (monocytes). **(e)** Quantification of CD11c$^+$ Ly6C$^-$ Ly6G$^-$ F480$^-$ CD64$^-$ MHC-II$^+$ cells within CD45$^+$ cells (dendritic cell types). **(f)** Representative myeloperoxidase (MPO) staining in colonic sections from WT and *Atg7*$^{\Delta IEC}$ mice before and after infection at day 8 and day 18 p.i. Quantification of MPO-positive cells of colonic sections (UI: n=6 WT, n=7 Atg7$^{\Delta IEC}$; at day 8 p.i: n=6 WT mice, n=6 *Atg7*$^{\Delta IEC}$ mice; at day 18 p.i: n=8 WT mice and n=8 *Atg7*$^{\Delta IEC}$ mice, from two independent experiments). Scale bar = 100µm. **(g)** Relative IL-17A and IL-17F mRNA level assessed by qRT-PCR on colonic tissue from uninfected (UI) WT and *Atg7*$^{\Delta IEC}$ mice and at day 8 and day 18 p.i. (UI: n=8 WT mice, n=9 *Atg7*$^{\Delta IEC}$ mice; at day 8 p.i: n=10 WT mice, n=8 *Atg7*$^{\Delta IEC}$ mice; at day 18 p.i: n=10 WT mice, n=11 *Atg7*$^{\Delta IEC}$ mice, two independent experiments). **(h)** Elisa analysis for IL-22 secretion in colon explant cultures of WT and *Atg7*$^{\Delta IEC}$ uninfected mice (UI: n=4 WT mice, n=4 *Atg7*$^{\Delta IEC}$). Significant difference: * $p<0.05$, ***$p<0.001$, ns = not significant, determined by unpaired *t*-test. Mean ± SEM.

Figure S5

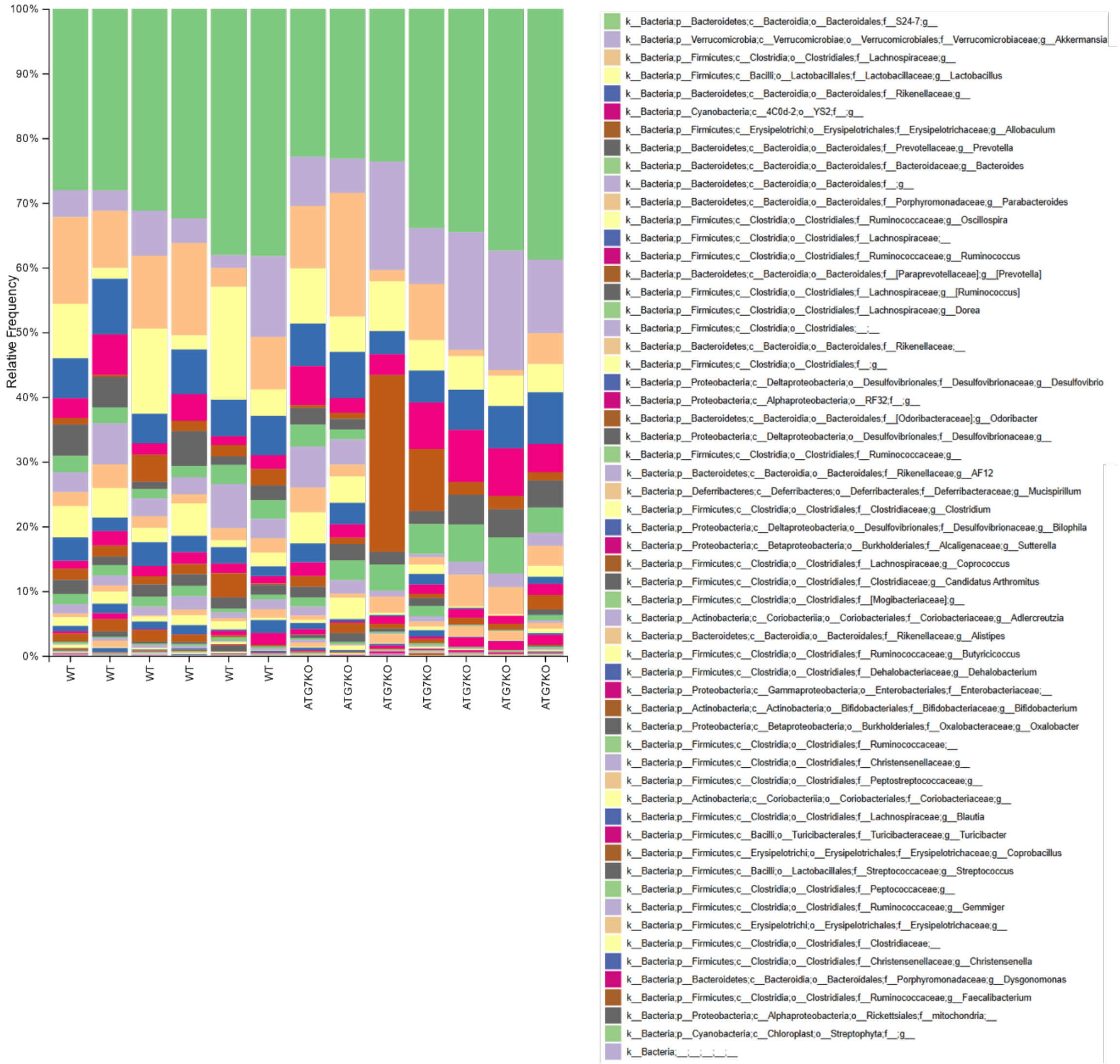

Fig S5: Effect of *Atg7* deletion on the microbiota composition. Taxonomic analysis of bacterial repartition in feces from WT and *Atg7*$^{\Delta IEC}$ mice (n=6 WT mice, n=6 *Atg7*$^{\Delta IEC}$ from two independent experiments).

Figure S6

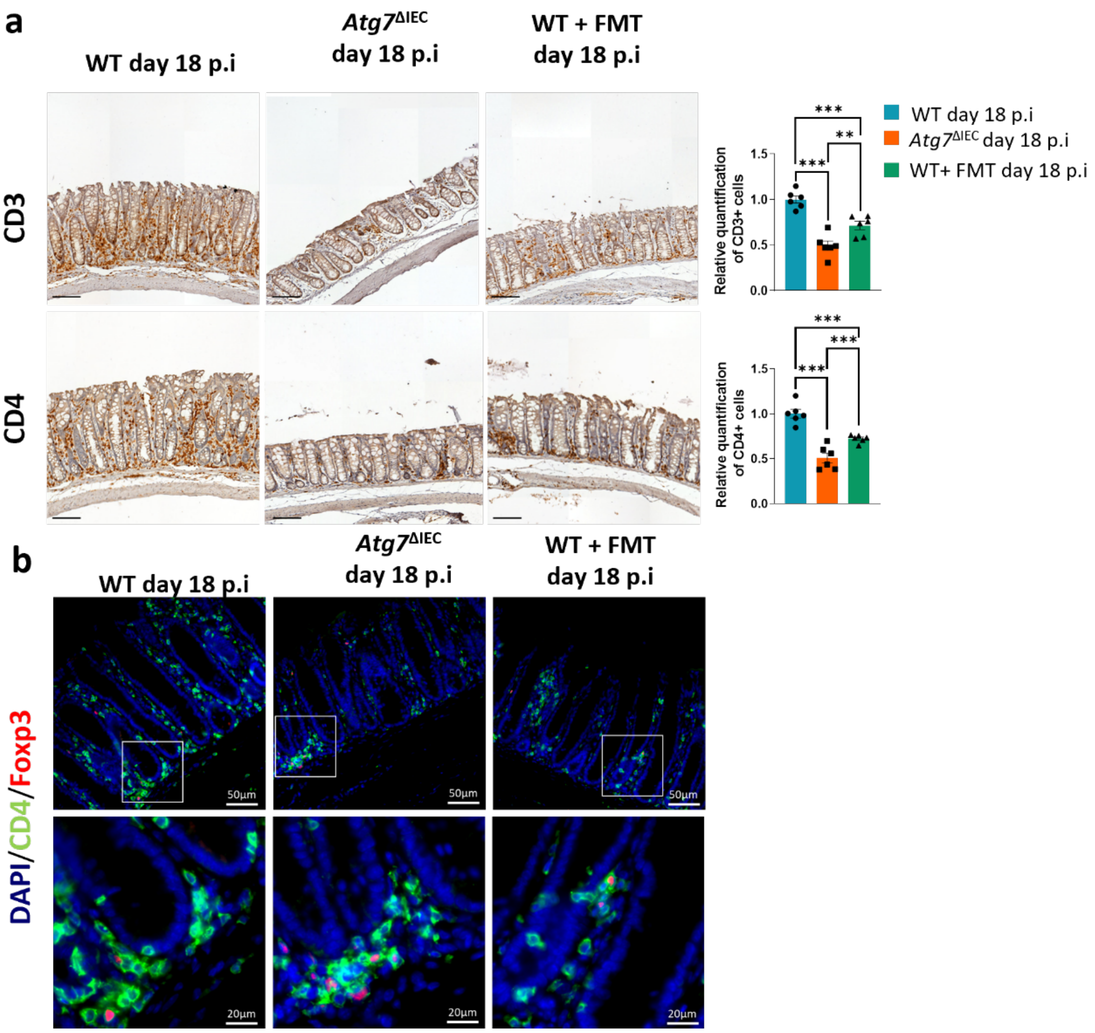

Fig S6: Effect of microbiota transplantation from *Atg7*-deficient mice on T cell recruitment in WT mice after *C. rodentium* infection. (a) Representative CD3 and CD4 stainings on colonic sections from WT, *Atg7*$^{\Delta IEC}$ and WT+FMT at day 18 p.i. Quantification of CD3 and CD4 (n=6 mice per condition, two independent experiments). Black scale bar= 100µm. **(b)** Representative IHC stainings for CD4 and Foxp3 on colonic sections from WT, *Atg7*$^{\Delta IEC}$ and WT+FMT mice at day 18 p.i. Significant difference: * p<0.05, ***p<0.001, ns = not significant, determined by unpaired *t*-test. Mean ± SEM.

Figure S7

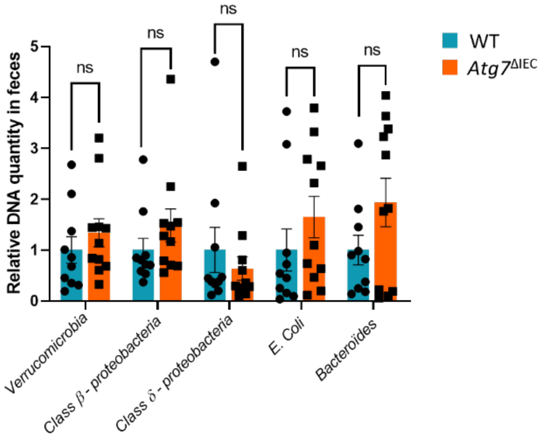

Fig S7: Effect of *Atg7* deletion on the prevalence of gram negative bacteria. Quantification of the abundance of *Verrucomicrobia,* class β - *proteobacteria,* class *δ* - *proteobacteria, E. Coli* and Bacteroïdes in WT and *Atg7*$^{\Delta IEC}$ fecal samples (n=10 WT and n=11 *Atg7*$^{\Delta IEC}$). ns = not significant, determined by unpaired *t*-test. Mean ± SEM.

Figure S8

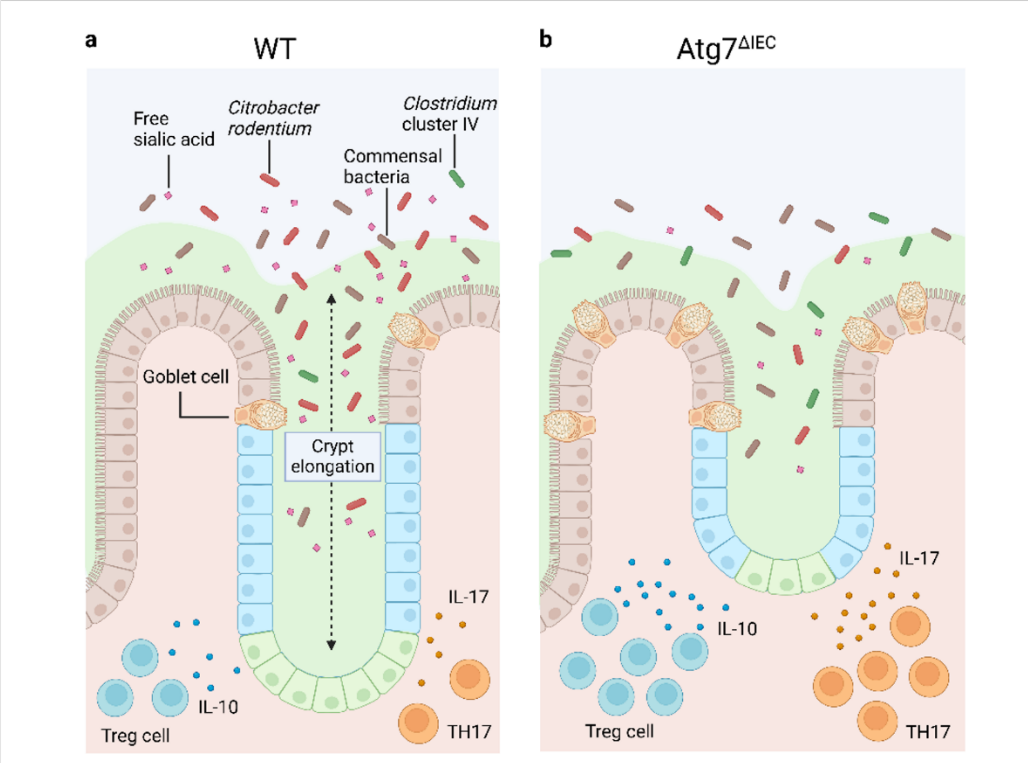

Fig S8: Model of Autophagy's Role in the Colon During C. rodentium Infection. **(a)** In the presence of autophagy, *C. rodentium* colonization is facilitated by free sialic acid, which acts as a nutrient. These sialic acids are released from glycans by commensal bacteria with sialidase activity. The invasion by *C. rodentium* triggers severe inflammation, crypt elongation, and significant recruitment of immune cells. **(b)** Loss of autophagy confers protection against *C. rodentium* infection by limiting inflammation, colonic epithelial dysplasia, and goblet cells loss. This protective effect is coupled to a higher proportion of IL-10-secreting Tregs and IL-17-secreting TH17 cells. These alterations in the immune response result from an Atg7-dependent changes in microbiota with increased proportion of Clostridium cluster IV and a decrease in microbiota with sialidase activity.
